# Hydridoorganostannylene Coordination: Group 4 Metallocene Dichloride Reduction in Reaction with Organodihydridostannate Anions

**DOI:** 10.1002/chem.201903652

**Published:** 2019-11-13

**Authors:** Jakob‐Jonathan Maudrich, Max Widemann, Fatima Diab, Ralf H. Kern, Peter Sirsch, Christian P. Sindlinger, Hartmut Schubert, Lars Wesemann

**Affiliations:** ^1^ Institut für Anorganische Chemie Universität Tübingen Auf der Morgenstelle 18 72076 Tübingen Germany; ^2^ Institut für Anorganische Chemie Georg-August Universität Göttingen Tammannstr 4 37077 Göttingen Germany

**Keywords:** abstraction, germanium, hydrides, metallocenes, tin

## Abstract

Organodihydridoelement anions of germanium and tin were reacted with metallocene dichlorides of Group 4 metals Ti, Zr and Hf. The germate anion [Ar*GeH_2_]^−^ reacts with hafnocene dichloride under formation of the substitution product [Cp_2_Hf(GeH_2_Ar*)_2_]. Reaction of the organodihydridostannate with metallocene dichlorides affords the reduction products [Cp_2_M(SnHAr*)_2_] (M=Ti, Zr, Hf). Abstraction of a hydride substituent from the titanium bis(hydridoorganostannylene) complex results in formation of cation [Cp_2_M(SnAr*)(SnHAr*)]^+^ exhibiting a short Ti–Sn interaction. (Ar*=2,6‐Trip_2_C_6_H_3_, Trip=2,4,6‐triisopropylphenyl).

## Graphical abstract

**Reaction of metallocene dichlorides** with anions of deprotonated organoelement trihydrides of germanium and tin affords substitution products or reduction followed by hydridoorganostannylene coordination.

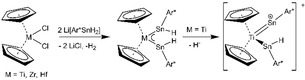

## Introduction

Since Lappert et al. invented the synthesis of germylenes and stannylenes in the early 1970s, the interaction of these Lewis basic and Lewis acidic molecules with transition metal fragments has been extensively studied.^1^ However, one of the first stannylene coordination compounds [Me_2_Sn(thf)Cr(CO)_5_] was already synthesized earlier, in 1971, following a salt metathesis reaction between dimethyl tin dichloride and the dinuclear chromium carbonylate dianion [Cr_2_(CO)_10_]^2−^.^2^ The coordination chemistry of diorgano‐ as well as diamidostannylenes became a very attractive field of research and was further developed for a variety of transition metal fragments. The nature of the bonding between low valent Group 14 elements and transition metals was also studied intensively. Diamidostannylenes, for example, act as donor ligands, whereas dialkylstannylenes can also act as acceptor ligands, by providing an empty p‐orbital.

The hydride chemistry of heavy Group 14 elements and, in particular, the chemistry of low valent elements have recently attracted major interest.^3^ Power et al. used bulky terphenyl ligands for the synthesis of the first low valent hydrides of tin and germanium.^4^ The coordination chemistry of low valent tin hydrides was investigated by Rivard et al. for the highly reactive SnH_2_, which was coordinated at transition metals to produce a Lewis base stabilized adduct.^5^ Tilley et al. reported the reaction of an osmium benzyl complex with organotin trihydride tripSnH_3_ (trip=2,4,6‐triisopropylphenyl) to give an organohydridostannylene complex upon toluene elimination.^6^ By reacting an NHC adduct of a low valent tin hydride [Ar*SnH(^Me^NHC)] with the platinum complex [Pt(cod)_2_] a dimeric tin–platinum complex featuring bridging hydride ligands was characterized at low temperature.^7^ We have been exploring the chemistry of organotin and organogermanium trihydrides: Both, reductive elimination of hydrogen in reaction with various Lewis bases, as well as hydride abstraction to give highly reactive dihydridocations were studied.^8^ Furthermore deprotonation with Brønsted bases, such as LiMe, LDA or KBn was investigated, which resulted in the formation of organodihydrido anions of germanium and tin.^9^

**Figure 1:**
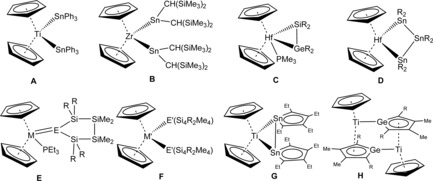
Group 14 element coordination at metallocenes of Group 4 metals. (M=Ti, Zr, Hf; E=Ge, Sn, Pb; M′=Ti, Zr; E′=Sn, Pb; R=SiMe_3_).

A large variety of Group 14 element ligands were coordinated at metallocene fragments of Group 4 metals.^10^ Nucleophilic substitution at metallocene dichlorides by using alkali metal salts of triphenyltin was reported as early as 1968 to yield the bis triphenyltin substitution product **A** (Figure 1).10a Piers et al. published the coordination of Lappert's stannylene [Sn{CH(SiMe_3_)_2_}_2_] at in situ generated zirconocene (**B**, Figure 1).10c, 10d, 11 This method was also used by Růžička and his group to coordinate a C,N‐chelated stannylene SnL_2_ [L=2‐{(dimethylamino)methyl}phenyl].10k Marschner and co‐workers synthesized a variety of Group 4 metallocene derivatives with Group 14 element ligands. A digermene or silagermene complex (**C**, Figure 1) of Group 4 metallocenes was synthesized by reaction of alkaline metal salts of digermanes or silagermanes with respective metallocene dichlorides.10e By reacting a comparable alkaline salt of a distannane and after stannylene transfer, a metallocene derivative exhibiting a four‐membered ring (**D**, Figure 1) was isolated.10e Coordination of cyclic tetrylenes at metallocene fragments was achieved by reacting the monomeric phosphine adduct of cyclic tetrylenes or a dimeric precursor of a cyclic stannylene or plumbylene with the in situ reduced metallocene (**E**, **F**, Figure 1).10f, 10g Saito et al. studied the reaction of dilithio stannole with titanocene dichloride and found formation of a TiSn_2_ moiety (**G** Figure 1).10h In comparison, dipotassium germole, which was investigated by Müller and co‐workers, was reacted with titanocene or zirconocene dichloride under elimination of potassium cyclopentadienide, which led to the formation of dimeric germole complexes (**H** Figure 1).10i Furthermore, diorganotin hydride coordination at hafnocene complexes was discussed, as an intermediate in catalytic dehydrocoupling of diorganostannanes.^12^ Here, we present the reaction of metallocene dichlorides of titanium, zirconium and hafnium with organodihydrido anions of germanium and tin.

## Results and Discussion

**Scheme 1:**
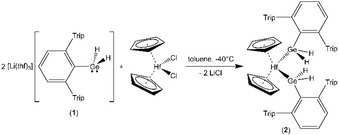
Synthesis of the hafnocene bis(dihydridogermyl) complex **2**.

Two equivalents of the lithium salt of organodihydridogermate salt **1** were reacted at −40 °C with hafnocene dichloride (Scheme 1). The substitution product **2**, which was isolated after crystallization from toluene, was characterized by NMR spectroscopy and elemental analysis. Crystals suitable for single crystal X‐ray diffraction, however, were not obtained. Germanium coordination at hafnium was previously observed for anionic germyl ligands [Ge(SiMe_3_)_3_],^13^ germylenes [Ge(Si*t*Bu_2_Me)_2_],^14^ [Ge(Si{SiMe_3_)_2_}_2_(SiMe_2_)_2_],10g a digermene [Ge(SiMe_3_)_2_]_2_,10e a silagermene (**C** Figure 1),10e and in germole chemistry.^15^

The signal for the GeH_2_ units in **2** was found at lower field (4.06 ppm) in comparison to the anionic starting material **1** (3.02 ppm) and the organogermanium trihydride Ar*GeH_3_ (3.61 ppm) (Ar*=2,6‐Trip_2_C_6_H_3_, Trip=2,4,6‐triisopropylphenyl). The organodihydridogermate salt **1** was also reacted with titanocene and zirconocene dichloride. In these reaction mixtures we were not able to characterize the reaction products.

**Scheme 2:**
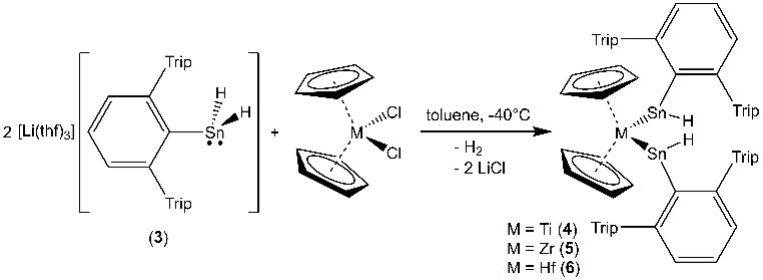
Synthesis of the metallocene bis(hydridoorgano‐stannylene) complexes **4**–**6**.

The homologous tin anion **3** was reacted with metallocene dichlorides of titanium, zirconium and hafnium (Scheme 2) to give the titanium bis(stannylene) complex **4** in high yield (96 %), whereas the zirconium and hafnium analogues were isolated in moderate yield (**5**: 69 %; **6**: 42 %).

**Figure 2:**
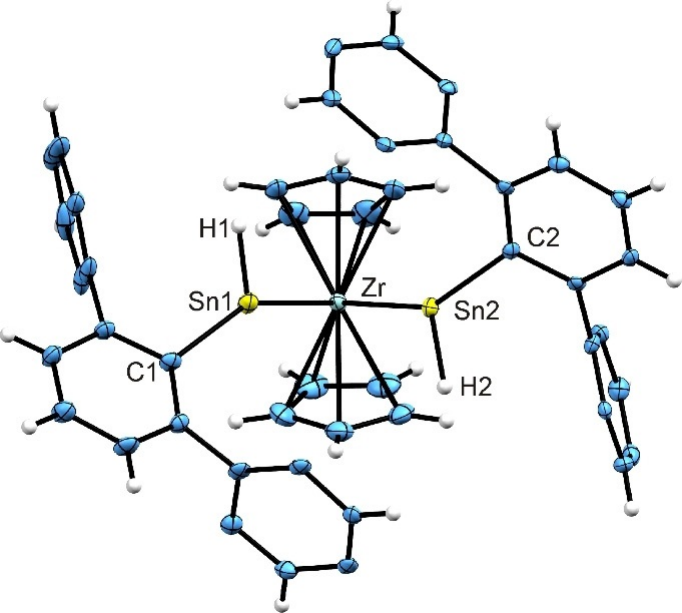
ORTEP for the molecular structure of **5**, with ellipsoids drawn at the 50 % probability level. Hydrogen atoms H1 and H2 were found in difference Fourier maps and freely refined, *i*Pr groups are omitted for clarity.

The metallocene complexes **4**–**6** were fully characterized by elemental analysis, NMR spectroscopy and single crystal X‐ray diffraction. Crystallographic data and refinement details were placed in the Supporting Information. Since the molecular structures of **4**–**6** display almost identical geometries in the solid state, only the molecular structure of **5** is shown in Figure 2, while representations of the molecular structures of **4** and **6** were placed in the Supporting Information. Selected interatomic distances and angles for all three systems are listed in Table 1. Despite the very bulky terphenyl substituent the Ti−Sn bond lengths in **4** [2.677(2), 2.686(2) Å] are slightly shorter than the distances found in other Ti−Sn complexes (see Figure 1): **E** 2.6940(9), **F** 2.7122(13), 2.7154(14), **G** 2.6867(16) and 2.7254(17) Å.10h, 16 In the case of the zirconium derivative **5** the Zr−Sn distances [2.795(1), 2.806(1) Å] are comparable with the Zr−Sn bond lengths found in **E** (2.7942(10) Å), but they are shorter than the values found in **B** (2.8715(11) Å) and in the complex Cp_2_Zr(SnL_2_)_2_ [2.8230(4)) and 2.8614(4) Å; L=2‐{(dimethylamino)methyl}phenyl].10c, 10d, 10f, 10k The Hf−Sn distances in **6** [2.770(1), 2.781(1) Å] are slightly longer than the bond length found in **E** (2.7585(11) Å). The Hf−Sn bond lengths in hafnocene derivatives [CpCp*Hf(SnPh_3_)  L], on the other hand, are significantly longer: L=Cl, 2.9650(4); NMe_2_, 2.9694(8); Me, 2.9740(5); OMe, 2.9556(5) Å.^17^ The Sn−Sn distances [**4**: 3.370(1), **5**: 3.526(1), **6**: 3.489(1) Å] in complexes **4**–**6** are longer than typical Sn−Sn single bonds [Ar′H_2_Sn‐SnH_2_Ar“, 2.7449(3)] [Ar′=2,6‐Mes_2_C_6_H_3_, Mes=2,4,6‐trimethylphenyl], however short enough to point towards a minor interaction between both tin atoms.8c Coordination of two stannylenes at metallocene fragments was previously reported in **B**, **F** and Cp_2_Zr(SnL_2_)_2_. The Sn−Sn distances in these derivatives are all clearly longer: 4.2364 (**B**), 3.804 (**F**‐Ti), 4.216 (**F**‐Hf) and 3.559 Å (Cp_2_Zr(SnL_2_)_2_). Therefore, it seems that the relatively small hydride substituent favors shorter interatomic Sn−Sn distances in **4**–**6**.

**Table 1:** Interatomic distances [Å] and angles [deg] in compounds **4**–**6**.

	**4** (Ti)	**5** (Zr)	**6** (Hf)
M−Sn1	2.677(2)	2.795(1)	2.770(1)
M−Sn2	2.686(2)	2.806(1)	2.781(1)
Sn1−Sn2	3.370(1)	3.526(1)	3.489(1)
Sn1−C1	2.208(8)	2.196(4)	2.198(4)
Sn2−C2	2.162(8)	2.198(4)	2.202(4)
Sn1−H1	1.69(2)	1.747(19)	1.776(18)
Sn2−H2	1.68(2)	1.743(19)	1.751(18)
M−Cp	2.349(8)–2.389(8)	2.452(5)–2.508(4)	2.429(4)–2.487(4)
Sn1‐M‐Sn2	77.8(1)	78.1(1)	77.4(1)
C1‐Sn1‐M	144.7(2)	144.7(1)	144.2(1)
C2‐Sn2‐M	143.5(2)	146.3(1)	144.9(1)

The coordinated organohydridostannylene Ar*SnH in complexes **4**–**6** display a downfield signal in the ^1^H NMR as well as in the ^119^Sn NMR spectrum (Table 2). Tilley et al. previously reported the stannylene complex [Cp*(*i*Pr_3_P)HOs=SnH(trip)], featuring a hydridostannylene SnH(trip) coordination. They observed the ^1^H NMR signal at 19.4 ppm and the ^119^Sn NMR signal at 786 ppm (^1^ *J*_Sn−H_ 775 Hz).^12^, ^18^ Diorganostannylenes and disilylated stannylenes coordinated at metallocene fragments also exhibit signals at low field in the ^119^Sn NMR (Figure 1: **B** 1677 ppm; **E** Ti 1635, Zr 1263, Hf 1080; **F** Hf 1785, Ti 2172 ppm).10g, 16

**Table 2:** NMR data of metallocene derivatives **4**–**6**.

Cp_2_M(Ar*SnH)_2_	^1^H NMR	^1^ *J*_Sn−H_	^119^Sn NMR
(**4**) Ti	13.27	750	1250
(**5**) Zr	12.49	750	1125
(**6**) Hf	12.63	890	1060

Complexes **4**–**6** show ^1^ *J*_Sn−H_ coupling constants in the range of 750–890 Hz (Table 2), which is comparable to the value observed for the aforementioned stannylene complex [Cp*(*i*Pr_3_P)HOs=SnH(trip)].

However, triply coordinated stannylene adducts Ar*SnH(L) (L=NHC, ^1^ *J*_Sn−H_ 237.5, 227.6 Hz; L=py, 99 Hz; L=DMAP, 113.2 Hz) (^Et^NHC=1,3‐diethyl‐4,5‐dimethylimidazol‐2‐ylidene, DMAP=4‐dimethylaminopyridine) show relatively small ^1^ *J*_Sn‐H_ coupling constants.8d, 8e Müller et al. have characterized related NHC‐stabilized hydridosilylenes [Ar′SiH(^Me^NHC)] and also identified small ^1^ *J*_SiH_ coupling constants, for example, 103 Hz [Ar′=2,6‐Mes_2_C_6_H_3_, Mes=2,4,6‐trimethylphenyl]. On the basis of quantum chemical calculations, the small coupling constant was shown to be a consequence of a strongly reduced Fermi contact and, accordingly, a small s‐orbital participation in the Si−H bond of only 16 %. This, in turn, was rationalized with the lone pair on the silicon atom residing in an orbital with mainly s character.^19^ Following this argumentation, the larger ^1^ *J*_Sn−H_ coupling constants in complexes **4**–**6** should be accompanied by a higher s‐orbital participation in the Sn−H bonds. In order to support this assumption we carried out DFT calculations on **4** and, for comparison, on the related complex [Ar*SnH(^Et^NHC)],^20^ and analyzed their bonding using the natural bond orbital (NBO) approach^21^ (for details, see the Supporting Information). In the NBO representing the Sn−H bonds in **4**, the tin atoms display 22.1 % s‐orbital participation. For comparison, in the adduct [Ar*SnH(^Et^NHC)] the 5s(Sn)‐orbital character of the Sn−H bond was determined to be 12.3 %. This supports the above interpretation and indicates a correlation between the magnitude of the ^1^ *J*_Sn−H_ coupling constant and s‐orbital participation of Sn in the Sn−H bond. However, it was shown for the higher homologue Pb, that the magnitude of ^1^ *J*(Pb−C) is not related to the s character of the Pb−C bond, as in this case relativistic effects play an important role.^22^

On the basis of the NBO analysis the bonding between the stannylene ligands and the titanocene fragment in **4** could be separated into two Sn−Ti σ‐bonds [each composed of 42 % Ti (16 % s, 84 % d character) and 58 % Sn (54 % s, 46 % p‐character) and a π‐bond between the Ti center and both Sn atoms [Ti: 48 % (97 % d character), Sn: 26 % each (99 % p‐character)], which is represented by a three‐centre NBO (for details, see the Supporting Information). The stannylene (Ar*SnH) therefore interacts with the titanocene fragment Cp_2_Ti as σ‐donor and π‐acceptor ligand, with the π‐backbonding from the Ti centre to the tin atoms distributed equally over both stannylene ligands. A similar bonding scenario was observed for the higher homologue Zr in the model system [Cp_2_Zr(SnHPh)_2_] (for details, see the Supporting Information), which confirms earlier findings by Piers, Marschner, Müller who have analyzed the bonding in bis(tetrylene) zirconocene complexes of type **B** and **F** (see Figure 1).10d, 10f Saito et al. reported a similar structural motif for their TiSn_2_‐ring complex (**G** in Figure 1). The nature of the bonding in this compound, however, is somewhat different and can be described by an electron donation from a σ(Sn−Sn) orbital towards Ti leading to a partial aromatic character of the TiSn2 ring.10h

Using the “Atoms in Molecules” (AIM) approach,^23^ the atomic charges in **4** were determined to be +1.25 for Ti and +0.68 for both Sn1 and Sn2. It is interesting to note that, due to their closeness, the π‐accepting p‐orbitals on both Sn atoms seem to overlap to a significant extent in the aforementioned three‐centre NBO, despite a slightly larger distance between both Sn atoms in the DFT‐optimized geometry of **4** (3.474 vs. 3.370(1) Å in the X‐ray diffraction study). A topological analysis of the electron density in the DFT model system for **4** revealed a relatively high delocalization index (DI) of 0.37 between both Sn atoms. The DI(A,B) is considered to measure the number of electron pairs shared between two atoms A and B, and represents the bond order in case of equal atoms.^24^ Therefore, this seems to point towards the existence of significant bonding interactions between the tin atoms in **4**. An even higher DI(Sn,Sn) of 0.52 was observed in the related DFT model system [Cp_2_Zr(SnHPh)_2_]. However, the Sn−Sn distance in this case was significantly shorter than in the X‐ray structure of **5**, probably due to the sterically less demanding stannylene ligands (3.273 vs. 3.526(1) Å).

**Scheme 3:**
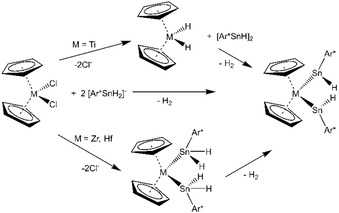
Two pathways for the formation of bis(hydridostannylene) metallocenes **4**–**6**.

Coordination of low valent Group 14 element ligands at metallocene fragments was so far accomplished by reaction with the alkyne complex [Cp_2_Ti(btmsa)]^25^ [btmsa: bis(trimethylsilyl)acetylene] or after reduction of the metallocene dichlorides with BuLi or Mg.10c, 10d, 10f, 10g, 11 Furthermore, the reaction of a dianionic main group compound, for example, dipotassiosilylgermane, with hafnocene dichloride also represents a method to coordinate a low valent compound, that is, a silagermane, to a metallocene.10e In the following, we discuss two possible pathways for the formation of the characterized metallocene complexes [Cp_2_M(SnHAr*)_2_] (**4**–**6**). Titanocene hydridostannylene [Cp_2_Ti(SnHAr*)_2_] (**4**) forms in a high yield (96 %) reaction. The reaction of Cp_2_TiCl_2_ with two equivalents of the anion [SnH_2_Ar*]^−^ was monitored by ^1^H NMR spectroscopy at room temperature. Immediately, formation of the organotin hydride [Ar*SnH]_2_ was observed making a hydride transfer to the titanocene complex feasible.4a However, hypothetically formed [Cp_2_TiH_2_] is highly reactive and should eliminate hydrogen (H_2_ was also detected in the ^1^H NMR spectrum) to give titanocene [Cp_2_Ti], as a further intermediate. This should then react directly with the low valent organotin hydride to give the isolated product **4** (Scheme 3).^26^

On the other hand, in the cases of zirconium and hafnium, we detected evolution of hydrogen by NMR spectroscopy and only slight amounts of organotin hydride [Ar*SnH]_2_. We assume that in these cases, substitution of the chloride ligands took place first, resulting in the formation of [Cp_2_M(SnH_2_Ar*)_2_], followed by evolution of hydrogen to give products **5** and **6** (Scheme 3). Potential dihydride complexes of Zr and Hf are known to be not as highly reactive as the homologous titanium dihydride.^27^ Since no formation of Cp_2_MH_2_ (M=Zr, Hf) was observed, we propose the mechanism shown here, without the formation of an intermediate dihydride.

**Scheme 4:**
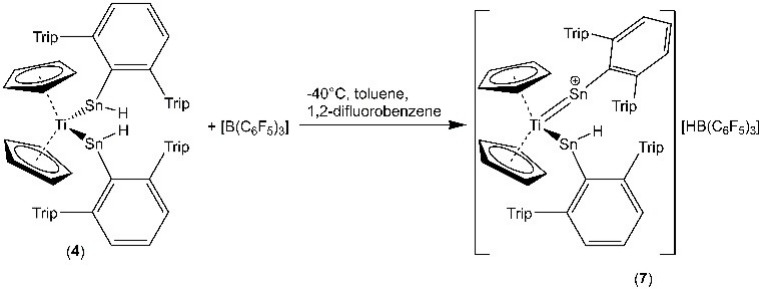
Hydride abstraction and synthesis of the cationic titanocene complex **7**.

Finally, the reactivity of the coordinated hydridostannylenes was investigated in the case of the titanium complex **4**. Hydride abstraction was studied by using the electrophile tris(pentafluorophenyl)borane (Scheme 4): In a 3:1 mixture of toluene and difluorobenzene at −40 °C, the borane [B(C_6_F_5_)_3_] was reacted with the stannylene complex Cp_2_Ti(SnHAr*)_2_ (**4**). The deep violet solution showed no significant color change after stirring at room temperature for 1 h. Crystals were obtained from a 1,2‐difluorobenzene solution by layering with hexane at −40 °C.

**Figure 3:**
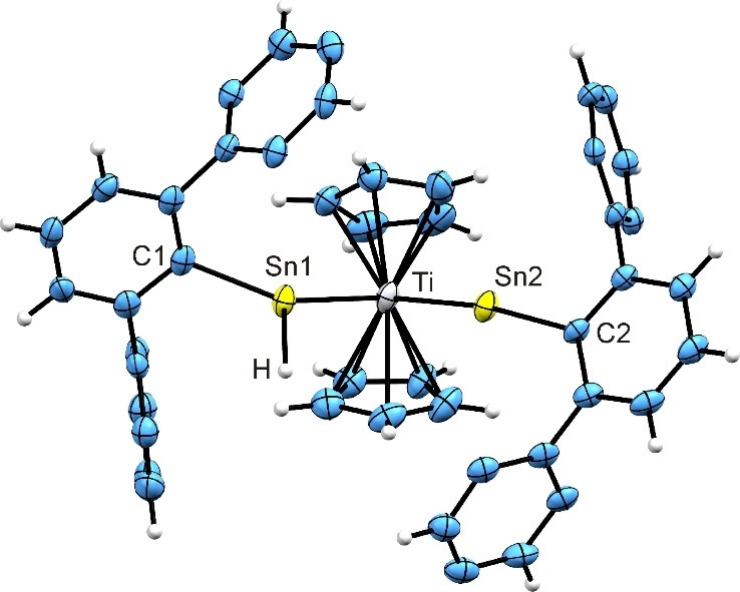
ORTEP for the molecular structure of **7**, with ellipsoids drawn at the 50 % probability level. The hydrogen atom attached to Sn1 was found in a difference Fourier map and freely refined, *i*Pr groups are omitted for clarity. Selected interatomic distances [Å] and angles [deg]: Sn1−C1 2.176(4), Sn2−C2 2.142(3), Ti−Sn1 2.6886(7), Ti−Sn2 2.5644(7), Sn1−H 1.887(17), Cp−Ti 2.318(4)‐2.400(4), Sn1−Sn2 3.925(1), Sn1‐Ti‐Sn2 96.7(1), C1‐Sn1‐Ti 145.0(1), C2‐Sn2‐Ti 167.4(1).

The Lewis acid [B(C_6_F_5_)_3_] reacted with bis(hydridoorganostannylene)titanocene complex **4** under abstraction of a hydride substituent and led to formation of a cationic complex **7**. In Figure 3 the molecular structure in the solid state is presented. The Ti‐Sn2 distance [2.5644(7) Å] of the cationic Ar*Sn moiety is shorter than the Ti−Sn1 distance [2.6886(7) Å] of the hydridostannylene ligand. For comparison, Ti−Sn complexes reported in literature exhibit longer interatomic distances: **E** 2.6940(9), **F** 2.7122(13), 2.7154(14), **G** 2.6867(16), 2.7254(17) Å (see Figure 1 for molecular schemes). To the best of our knowledge, the observed Ti−Sn2 bond length is the shortest Ti−Sn bond characterized so far.10h, 16 Moreover, upon hydride abstraction the Ti‐Sn2‐C2 angle [167.4(1)°] exhibits a linearization of 23° in comparison to the bis(stannylene) complex **4**.

Complexes with bent or linear M‐E‐R (M=transition metal, E=Group 14 element, R=organic substituent) arrangements were investigated by Power et al. and Filippou et al.^28^ Depending on the bond order between transition metal and low valent Group 14 element, either a linear metal tetrylidyne, M≡E‐R, or a metallo tetrylene, M‐E‐R, with a bent geometry, was found.^18^, ^28^, ^29^

**Figure 4:**
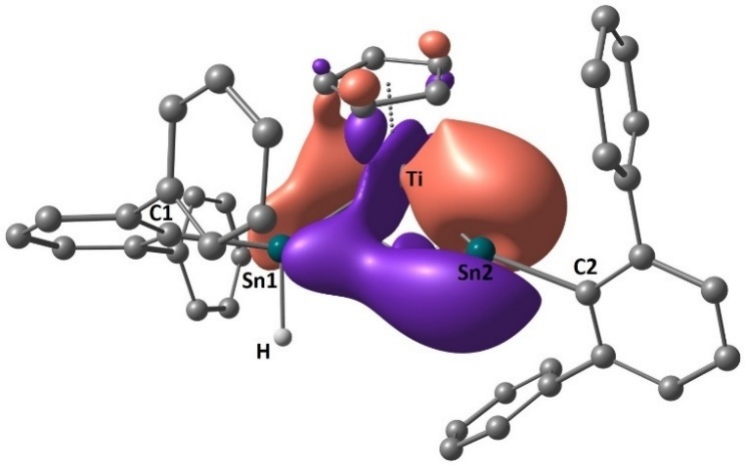
DFT optimized geometry of **7**, superimposed with the NLMO representing the back‐bonding from a filled titanium d‐orbital into the empty p‐orbitals of tin atoms Sn1 and Sn2.^30^

To explore the nature of the bonding in the cationic complex **7**, quantum chemical calculations were carried out. The DFT‐optimized geometry of **7** (Figure 4) was in good agreement with the solid‐state structure (for details, see the Supporting Information). An NBO analysis revealed that both tin ligands exhibit a Ti−Sn σ‐bond. In addition, a π‐interaction between the titanium atom and Sn2 was identified (for figures and details, see the Supporting Information), however with a clearly reduced occupation (1.64) of the respective NBO. This indicates a significant delocalization towards other atoms in the complex. The corresponding natural localized molecular orbital (NLMO) revealed that the atom Sn1 of the hydridostannylene ligand also receives some π‐bonding from the Ti centre, although to a much smaller extent than Sn2 (see Figure 4). Therefore, only 6.2 % of the electrons reside on Sn1, whereas 25.5 % are found on Sn2; the Ti centre comprises 56 % of the electrons. This suggests that due to the higher electrophilicity of the cationic Ar*Sn ligand, tin atom Sn2 abstracts a higher amount of the available electron pair from the titanium atom into its empty p‐orbital. The higher degree of π‐bonding then leads to a clearly shorter Ti−Sn2 bond and a larger angle C2‐Sn2‐Ti. This interpretation is also corroborated by the values for the delocalization index (DI), which can be obtained from the electron density distribution: For Ti−Sn1 and Ti−Sn2 a DI of 0.53 and 0.77, respectively, was calculated. For comparison, in the neutral complex **4**, a DI(Ti−Sn) of 0.70 was obtained, which again demonstrates a shift of the π‐bonding towards Sn2 upon abstraction of the hydride substituent. It is interesting to note that the increased back‐bonding leads to a smaller atomic charge (using the AIM approach) on Sn2, when compared to Sn1 (+0.80 vs. +0.95; Ti: +1.40). Finally, the remaining empty p‐orbitals of both tin atoms are part of the LUMO, LUMO+1 and LUMO+2 orbitals (for details, see the Supporting Information).

The signal of the tin hydride proton of **7** was found at low field (16.88 ppm, ^1^ *J*_Sn−H_=550 Hz) exhibiting a reduction of the Sn−H coupling constant in comparison to the starting material (**4**: 13.27 ppm, ^1^ *J*_Sn−H_=750 Hz). This low‐field shift might be due to the cationic character of **7**. For comparison, osmium complex [Cp*(*i*Pr_3_P)HOs=SnH(trip)] showed a ^1^H NMR signal for the Sn−H moiety at 19.4 ppm. In this example, the low‐field shift could be explained with the influence of the heavy atom on the light atom chemical shift.^31^

In the ^119^Sn NMR spectrum of **7**, two signals, a doublet (1484 ppm, ^1^ *J*_119Sn−H_=550 Hz) and a singlet (1789 ppm), were found. The cationic Ar*Sn moiety exhibits the signal at lower field, compared to the signal of the coordinated stannylene. The chemical shift of the coordinated stannylene lies close to the signal of the starting material **4** at 1250 ppm. On the basis of the optimized geometry of cation **7**, NMR calculations were performed using ADF with the implemented GGA revPBE‐D3(BJ) functional and ZORA TZ2P basis set.^32^ The obtained values are in excellent accordance with the measured values: Sn1 1484 (1425); Sn2 1789 (1754) ppm (calculated chemical shift in brackets).

Among a series of metal stannylidyne complexes, Filippou et al. also reported a synthesis, in which a manganese chlorostannylidene complex was transformed into a stannylidyne complex by abstraction of the chloride substituent from the coordinated chloridoorganostannylene.28m For the synthesis of the Ti‐Sn‐Ar* moiety we presented here, we employed a closely related approach, by abstracting a hydride substituent from a coordinated hydridoorganostannylene. In contrast to Filippou et al., however, we did not observe formation of a Ti−Sn triple bond, although another empty p‐orbital would be available at the cationic tin substituent. This can be explained by the fact that a further electron pair is missing in the Cp_2_Ti^II^ fragment, which would be necessary for increased backdonation. Therefore, hydride abstraction only leads to formation of a partial double bond between the titanium centre and the cationic tin atom.

## Conclusion

In contrast to an organodihydridogermate anion, which reacts under nucleophilic substitution with hafnocene dichloride, the homologous organodihydridostannate anion reacts as a reducing agent with Group 4 metallocene dichlorides. The formed metallocenes of titanium, zirconium and hafnium were stabilized by hydridostannylene coordination. Hydridoorganostannylene ligands coordinate via a Sn−M σ‐bond and both Sn atoms share the π‐backdonation of the metal electron pair into the empty tin p‐orbitals. Hydride abstraction from a hydridoorganotin complex of titanocene represents a synthetic pathway to increase the bond order of the Ti−Sn interaction.

## Conflict of interest

The authors declare no conflict of interest.

## Supporting information

As a service to our authors and readers, this journal provides supporting information supplied by the authors. Such materials are peer reviewed and may be re‐organized for online delivery, but are not copy‐edited or typeset. Technical support issues arising from supporting information (other than missing files) should be addressed to the authors.

Supplementary
