# Supplementary material for: Hydridoorganostannylene Coordination: Group 4 Metallocene Dichloride Reduction in Reaction with Organodihydridostannate Anions
Source: Chemistry. 2019 Nov 13;25(70):16081–7. doi: 10.1002/chem.201903652 (PMC6972602; doi:10.1002/chem.201903652)
Supplement: Supplementary file 1 — Supplementary [file CHEM-25-16081-s001.pdf]

CHEMISTRY
A European Journal

Supporting Information

Hydridoorganostannylene Coordination: Group 4 Metallocene Dichloride Reduction in Reaction with Organodihydridostannate Anions

Jakob-Jonathan Maudrich,[a] Max Widemann,[a] Fatima Diab,[a] Ralf H. Kern,[a] Peter Sirsch,[a] Christian P. Sindlinger,[b] Hartmut Schubert,[a] and Lars Wesemann*[a]

chem_201903652_sm_miscellaneous_information.pdf

Supporting Information

Hydridoorganostannylene coordination - Group 4 metallocene dichloride reduction in reaction with organodihydrostannate anions

Jakob-Jonathan Maudrich, Max Widemann, Fatima Diab, Ralf H. Kern, Peter Sirsch, Christian P. Sindlinger, Hartmut Schubert, Lars Wesemann*

Content

1. Experimental section 2
2. Crystal structure analysis 4
3. NMR data 8
   3.1 NMR spectra of compound **2** 8
   3.2 NMR spectra of compound **4** 10
   3.3 NMR spectra of compound **5** 13
   3.4 NMR spectra of compound **6** 16
   3.5 NMR spectra of compound **7** 19
4. IR spectra 23
5. Quantum chemical calculations 28
6. References 35

1. Experimental section

All manipulations were carried out under an argon atmosphere using standard Schlenk techniques or an MBraun Glovebox. Tetrahydrofurane and toluene were distilled from potassium, while *n*-hexane was obtained from an MBRAUN solvent purification system. Benzene-d_6 was also distilled from sodium. In addition, all solvents were repeatedly degassed by several freeze-pump-thaw cycles and stored in a glovebox. $[Li(thf)_3][2,6\text{-}Trip_2C_6H_3GeH_2]$ and $[Li(thf)_3][2,6\text{-}Trip_2C_6H_3SnH_2]$ were synthesized following literature procedures.[1] All other compounds were purchased commercially (Aldrich) and used without further purification. Elemental analysis was performed by the Institut für Anorganische Chemie, Universität Tübingen using a Vario MICRO EL analyzer.

NMR spectroscopy: NMR spectra were recorded on a Bruker AVIII-300 NanoBay spectromter (^{1}H 300.13 MHz; ^{13}C 75.47 MHz; ^{119}Sn 111.92 MHz) equipped with a 5 mm BBFO probe head, a Bruker AvanceII+400 NMR spectrometer (^{1}H 400.11 MHz; ^{13}C 100.61 MHz) equipped with a 5 mm QNP (quad nucleus probe) head or a Bruker AvanceII+500 NMR-spectrometer (^{1}H 500.13 MHz; ^{13}C 125.76 MHz; ^{119}Sn 186.50 MHz) equipped with a 5 mm ATM or a 5 mm TBO probe head and a setup for variable temperature. Routinely, "room temperature" NMR spectra were obtained at 26°C. The chemical shifts are reported in δ values in ppm relative to external $SiMe_4$ (^{1}H, ^{13}C) or $SnMe_4$ (^{119}Sn) using the chemical shift of the solvent ^{2}H resonance frequency and Ξ = 25.145020% for ^{13}C and 37.290632% for ^{119}Sn.[42] The multiplicity of the signals is abbreviated as s = singlet, d = doublet, t = triplet, quint = quintet, sept = septet and m = multiplet or unresolved. The proton and carbon signals were assigned by detailed analysis of ^{1}H, ^{13}C{^{1}H}, ^{1}H-^{1}H COSY, ^{1}H-^{13}C HSQC, ^{1}H-^{13}C HMBC and ^{13}C{^{1}H} DEPT-135 NMR spectra.

IR spectroscopy: IR spectra were recorded as KBr pellets prepared in a glovebox and measured with a Bruker VERTEX 70 IR spectrometer.

Syntheses:

[$Cp_2Hf(GeH_2Ar^*)_2$] (2). At rt a solution of $Li(thf)_3Ar^*GeH_2$ (**1**) (30 mg, 0.038 mmol, 2 equiv) in toluene (0.25 mL) was added at once to a suspension of Cp_2HfCl_2 (7.3 mg, 0.019 mmol, 1 equiv) in toluene (0.25 mL). The dark orange and turbid mixture was stirred for 30 minutes at room temperature and filtered through a syringe filter. Volatiles were removed under reduced pressure. $Cp_2Hf(GeH_2Ar^*)_2$ was obtained as dark orange powder (26.3 mg, 0.018, 96 %; purity > 95 %). Crystals, which were not suitable for X-ray diffraction, were obtained from saturated toluene solutions at –40°C. **^{1}H-NMR (**C_6D_6, 400.13 Hz): δ (ppm) 7.25 (s, 4H, m-C*H*$_{Trip}$), 7.18-7.10 (m, 3H, m-, p- C*H*$_{TripPh}$), 5.25 (s, 5H, C_5H_5), 4.06 (s, 2H, Ge*H*$_2$), 2.97 (sept, 2H, *p*-C*H*$(CH_3)_2$, $^3J_{HH}$ = 6.9Hz), 2.86 (sept, 4H, *o*-C*H*$(CH_3)_2$, $^3J_{HH}$ = 6.9Hz), 1.38 (d, 12H, p-CH$(CH_3)_2$, $^3J_{HH}$ = 6.9Hz), 1.36 (d, 12H, o-CH$(CH_3)_2$, $^3J_{HH}$ = 6.9Hz), 1.09 (d, 12H, o-CH$(CH_3)_2$, $^3J_{HH}$ = 6.9Hz). **^{13}C{^{1}H}-NMR** (C_6D_6, 125.75 Hz): δ (ppm) 151.2 (Ge-C_{TripPh}), 148.1 (*p*-C_{Trip}), 147.0 (*o*-C_{Trip}), 146.5 (*o*-C_{TripPh}), 140.2 (*ipso*-C_{Trip}), 129.0 (*p*-C_{TripPh}), 125.3(*m*-C_{TripPh}), 120.7 (*m*-C_{Trip}), 106.9 (C_5H_5), 34.7 (*p*-*C*H$(CH_3)_2$), 31.0 (*o*-*C*H$(CH_3)_2$), 26.0 (*o*-*C*H$(CH_3)_2$), 24.4 (*p*-*C*H$(CH_3)_2$), 23.0 (*o*-*C*H$(CH_3)_2$). ν Ge–H 1604 cm^{-1}. Anal. calcd. for $C_{82}H_{112}Sn_2Hf\cdot C_7H_8$: C 70.62, H 7.99; found: C 70.47, H 7.87.

[$Cp_2Ti(SnHAr^*)_2$] (4). A cooled (−40 °C) solution of $Li(thf)_3Ar^*SnH_2$ (150.0 mg, 0.182 mmol, 2.00 equiv) in THF (4 mL) was added dropwise to a solution of Cp_2TiCl_2 (23.3 mg, 0.0908 mmol, 1.00 equiv) in THF (3 mL). The resulting, deep red mixture is stirred for 20 hours at room temperature. After removal of the solvent, the deep violet residue is extracted with toluene (25 mL). Removal of the solvent yields $Cp_2Ti(SnHAr^*)_2$ as a deep violet solid, which is stored at −40 °C to avoid decomposition (120.8 mg, 0.0875 mmol, 96 %). Crystals suitable for X-ray analysis could be obtained from a saturated solution in THF. Analytical data for $Cp_2Ti(SnHAr^*)_2$: **^{1}H NMR** (400.11 MHz, C_6D_6): δ (ppm) 13.27 (s+broad satellites, 2H, $^1J_{119/117Sn\text{-}H}$=750 Hz, Sn*H*), 7.27 – 7.33 (m, 4H, *m*-C_6H_3), 7.24 (s, 4H, *m*-C_6H_2), 7.17 – 7.23 (m, 2H, *p*-C_6H_3), 7.15 (s, 4H, *m*-C_6H_2,

superimposed by solvent signal), 4.42 (s, 10H, $C_5\mathit{H}_5$), 3.22 (br sept, 4H, $^3J_{H\text{-}H}$ = 6.7 Hz, *o*-C*H*Me$_2$), 2.98 (br sept, 4H, *o*-C*H*Me$_2$), 2.93 (sept, 4H, $^3J_{H\text{-}H}$ = 6.9 Hz, *p*-C*H*Me$_2$), 1.61 (d, 12H, $^3J_{H\text{-}H}$ = 6.8 Hz, *o*-CHMe_2), 1.35 (d, 12H, $^3J_{H\text{-}H}$ = 6.9 Hz, *p*-CHMe_2), 1.33 (d, 12H, $^3J_{H\text{-}H}$ = 6.9 Hz, *p*-CHMe_2), 1.28 (br, 12H, $^3J_{H\text{-}H}$ = 6.8 Hz, *o*-CHMe_2), 1.24 (d, 12H, $^3J_{H\text{-}H}$ = 6.8 Hz, *o*-CHMe_2), 1.09 (d, 12H, $^3J_{H\text{-}H}$ = 6.9 Hz, *o*-CHMe_2). **^{13}C{^{1}H} NMR** (150.90 MHz, C_6D_6): δ (ppm) 167.4 (*C*–Sn), 147.3 (*o*-C_{Trip}), 146.6 (*p*-C_{Trip}), 144.7 (*o*-C_6H_3), 145.1 (*o*-C_{Trip}), 183.4 (br s, ipso-C_{Trip}), 129.7 (*m*-C_6H_3), 124.9 (*p*-C_6H_3), 120.8 (*m*-C_{Trip}), 119.7 (*m*-C_{Trip}), 90.6 (C_5H_5), 34.0 (*p*-*C*HMe$_2$), 30.1 (*o*-*C*HMe$_2$), 30.0 (*o*-*C*HMe$_2$), 26.0 (*o*-CHMe_2), 25.0 (*o*-CHMe_2), 24.0 (*p*-CHMe_2), 23.0 (*o*/*p*-CHMe_2), 21.4 (*o*-CHMe_2). **^{119}Sn NMR** (93.28 MHz, THF-*d8*): δ [ppm] 1250 (d, $^1J_{119Sn\text{-}H}$ = 750 Hz, *Sn*H). ν Sn–H 1741 cm^{-1}. Anal. calcd. for $C_{82}H_{110}Sn_2Ti$: C 71.31, H 8.03; found: C 71.23, H 7.98.

[$Cp_2M(SnHAr^*)_2$] [M = Zr (5), Hf (6)]. A solution of Li(thf)$_3$Ar*SnH$_2$ (40 mg, 0.048 mmol, 2 equiv) in toluene (0.2 mL) was added at once to a suspension of Cp_2MCl_2 (M=Zr: 7.2 mg, 0.024 mmol, 1 equiv; M=Hf: 9.2 mg, 0.024 mmol, 1 equiv) in toluene (0.2 mL). The deep red and turbid mixture is stirred for 30 minutes at room temperature and filtered through a syringe filter. Pentane was allowed to diffuse into the reaction mixture over a period of 2−3 days at room temperature. The supernatant solution was decanted and the crystals were washed with pentane (3×0.3 mL) and dried in vacuo to yield $Cp_2M(SnHAr^*)_2$ (M = Zr: deep violet crystals, 23.7 mg, 0.017 mmol, 69 %; M=Hf: deep red crystals, 15.3 mg, 0.010 mmol, 42 %).

Analytical data for [$Cp_2Zr(SnHAr^*)_2$]: **^{1}H NMR** (500.13 MHz, C_6D_6): δ (ppm) 12.49 (s+broad satellites, 2H, $^1J_{119Sn\text{-}H}$=750 Hz, Sn*H*), 7.29 (d, 4H, $^3J_{H\text{-}H}$=7.4 Hz, *m*-C_6H_3), 7.22-7.17 (m, 10H, *p*-C_6H_3, $C_6\mathit{H}_2$), 4.76 (s, 10H, C_5H_5), 3.26 (br sept, 4H, $^3J_{H\text{-}H}$=6.7 Hz, *o*-C*H*Me$_2$), 3.00 (br sept, 4H, $^3J_{H\text{-}H}$=6.6 Hz, *o*-C*H*Me$_2$), 2.93 (sept, 4H, $^3J_{H\text{-}H}$=7.0 Hz, *p*-C*H*Me$_2$), 1.57 (d, 12H, $^3J_{H\text{-}H}$=6.6 Hz, *o*-CHMe_2), 1.37 (d, 12H, $^3J_{H\text{-}H}$=6.7 Hz, *o*-CHMe_2), 1.34 (d, 12H, $^3J_{H\text{-}H}$=7.0 Hz, *p*-CHMe_2), 1.32 (d, 12H, $^3J_{H\text{-}H}$=7.0 Hz, *p*-CHMe_2), 1.24 (d, 12H, $^3J_{H\text{-}H}$=6.6 Hz, *o*-CHMe_2), 1.11 (d, 12H, $^3J_{H\text{-}H}$=6.7 Hz, *o*-CHMe_2). **^{13}C{^{1}H} NMR** (125.76 MHz, C_6D_6): δ (ppm) 165.6 (*C*–Sn), 148.0 (*p*-C_{Trip}), 147.1 (*o*-C_6H_3), 146.0 (*o*-C_{Trip}), 139.7 (*i*-C_{Trip}), 130.3 (*m*-C_6H_3), 125.9 (*p*-C_6H_3), 121.6 (*m*-C_{Trip}), 120.6 (*m*-C_{Trip}), 94.9 (C_5H_5), 34.8 (*p*-*C*HMe$_2$), 30.8 (*o*-*C*HMe$_2$), 30.7 (*o*-*C*HMe$_2$), 26.6 (*o*-CHMe_2), 25.8 (*o*-CHMe_2), 24.7 (*p*-CHMe_2), 23.9 (*p*-CHMe_2), 23.9 (*o*-CHMe_2), 22.5 (*o*-CHMe_2). **^{119}Sn NMR** (186.50 MHz, C_6D_6): δ (ppm) 1125 (d, $^1J_{119Sn\text{-}H}$=750 Hz, *Sn*H). ν Sn–H 1743 cm^{-1}. Anal. calcd. for $C_{82}H_{110}Sn_2Zr\cdot C_7H_8$: C 70.49, H 7.84; found: C 70.04, H 7.76.

Analytical data for [$Cp_2Hf(SnHAr^*)_2$]: **^{1}H NMR** (500.13 MHz, C_6D_6): δ (ppm) 12.63 (s+broad satellites, 2H, $^1J_{Sn\text{-}H}$ = ca. 830 Hz, Sn*H*), 7.29 (d, 4H, $^3J_{H\text{-}H}$=7.4 Hz, *m*-$C_6\mathit{H}_3$), 7.22−7.17 (m, 10H, *p*-$C_6\mathit{H}_3$, $C_6\mathit{H}_2$), 4.69 (s, 10H, $C_5\mathit{H}_5$), 3.27 (sept, 4H, $^3J_{H\text{-}H}$=6.7 Hz, *o*-C*H*Me$_2$), 2.98 (sept, 4H, $^3J_{H\text{-}H}$ = 6.8 Hz, *o*-C*H*Me$_2$), 2.92 (sept, 4H, $^3J_{H\text{-}H}$=7.0 Hz, *p*-C*H*Me$_2$), 1.55 (d, 12H, $^3J_{H\text{-}H}$ = 6.8 Hz, *o*-CHMe_2), 1.37 (d, 12H, $^3J_{H\text{-}H}$ = 6.8 Hz, *o*-CHMe_2), 1.33 (d, $^3J_{H\text{-}H}$ = 7.0 Hz, *p*-CHMe_2), 1.31 (d, $^3J_{H\text{-}H}$ = 6.9 Hz, *p*-CHMe_2), 1.24 (d, 12H, $^3J_{H\text{-}H}$ = 6.7 Hz, *o*-CHMe_2), 1.10 (d, 12H, $^3J_{H\text{-}H}$ = 6.7 Hz, *o*-CHMe_2). **^{13}C{^{1}H} NMR** (125.76 MHz, C_6D_6): δ (ppm) 165.8 (*C*-Sn), 147.9 (*p*-C_{Trip}), 147.0 (*o*-C_{Trip}), 146.4 (*o*-C_6H_3), 145.9 (*o*-C_{Trip}), 140.2 (*i*-C_{Trip}), 130.4 (*m*-C_6H_3), 125.7 (*p*-C_6H_3), 121.5 (*m*-C_{Trip}), 120.7 (*m*-C_{Trip}), 94.0 (C_5H_5), 34.8 (*p*-*C*HMe$_2$), 30.8 (*o*-*C*HMe$_2$), 30.7 (*o*-*C*HMe$_2$), 26.6 (*o*-CHMe_2), 25.7 (*o*-CHMe_2), 24.7 (*p*-CHMe_2), 23.9 (*o*-CHMe_2), 23.8 (*p*-CHMe_2), 22.5 (*o*-CHMe_2). **^{119}Sn NMR** (186.50 MHz, C_6D_6): δ (ppm) 1060 (d, $^1J_{119Sn\text{-}H}$ = ca. 890 Hz, *Sn*H). ν Sn–H 1749 cm^{-1}. Anal. calcd. for $C_{82}H_{110}Sn_2Hf$: C 65.15, H 7.33; found: C 65.34, H 7.06.

[$Cp_2Ti(SnAr^*)(SnHAr^*)$][$HB(C_6F_5)_3$] (7). A cooled (−40°C) solution of tris(pentafluorophenyl)borane (27.8 mg, 0.0542 mmol, 1.00 equiv) in toluene (1.5 mL) and 1,2-difluorobenzene (0.5 mL) was added dropwise to a cooled (−40°C) solution of $Cp_2Ti(SnHAr^*)_2$ (74.9 mg, 0.0542 mmol, 1.00 equiv) in toluene (2 mL) and 1,2-difluorobenzene (0.5 mL). The deep violet solution was stirred for 1 h at room temperature and the solvents were removed in vacuo. The dark residue was redissolved in 1,2-difluorobenzene (ca. 0.5 mL), layered with hexane (ca. 2-3 mL) and stored at −40°C. After complete diffusion the supernatant solution was decanted and the crystals were washed with cold hexane (3×0.5 mL). Drying in vacuo yields [$Cp_2Ti(SnAr^*)(SnHAr^*)$][$HB(C_6F_5)_3$] as a dark violet, crystalline solid, which is stored at −40 °C to avoid decomposition (68.2 mg, 0.0360 mmol, 66 %). Analytical data for [$Cp_2Ti(SnAr^*)(SnHAr^*)$][$HB(C_6F_5)_3$]: **^{1}H NMR** (400.11 MHz, C_6D_6 + 1,2-Difluorobenzene): δ (ppm) 16.88 (s + Satelliten, $^1J_{119/117Sn\text{-}1H}$ = 550 Hz, 1H,

Sn*H*), 7.23 (s, 4H, *m*-C_6H_2), 7.16 – 7.21 (m, 4H, *m*/*p*-C_6H_3), 7.16 (s, 4H, *m*-C_6H_2), 7.09 (d, 2H, $^3J_{HH}$ = 7.4 Hz, *m*-C_6H_3), 4.20 (s, 10H, C_5H_5), 2.91 (sept, 2H, $^3J_{HH}$ = 6.9 Hz, *o*/*p*-C*H*Me₂), 2.79 – 2.90 (m, 6H, *o*/*p*-*o*-C*H*Me_2), 2.78 (sept, 4H, $^3J_{HH}$ = 6.9 Hz, *o*/*p*-*o*-C*H*Me_2), 1.30 (d, 12H, $^3J_{HH}$ = 6.9 Hz, *o*/*p*- CH*Me*₂), 1.23 (d, 12H, $^3J_{HH}$ = 6.8 Hz, *o*/*p* -CHMe_2), 1.22 (d, 12H, $^3J_{HH}$ = 6.9 Hz, *o*/*p* -CHMe_2), 1.07 – 1.26 (br, 12H, *o*/*p* -CHMe_2), 1.01 (d, 12H, $^3J_{HH}$ = 6.8 Hz, *o*/*p* -CHMe_2), 0.98 (d, 12H, $^3J_{HH}$ = 6.8 Hz, *o*/*p*-CHMe_2). **^{13}C{^{1}H} NMR** (100.61 MHz, C_6D_6 + 1,2-Difluorbenzene): δ (ppm) 191.1 (Sn-C_6H_3), 167.0 (H-Sn-C_6H_3), 150.3 (*o*/*p*-C_{Trip}), 149.2 (superimposed by solvent signal of ODF, *o*/*p*-C_{Trip}), 148.8 (br d, $^1J_{19F-13C}$ = 240 Hz, *C*F), 148.1 (*o*/*p*-C_{Trip}), 144.0 (*o*-C_6H_3), 138.8 (*o*-C_6H_3), 138.0 (br d, $^1J_{19F-13C}$ = 250 Hz, *C*F), 136.8 (br d, $^1J_{19F-13C}$ = 250 Hz, *C*F), 135.2 (ipso-C_{Trip}), 133.5 (ipso-C_{Trip}), 131.2 (*m*-C_6H_3), 130.9 (*m*-C_6H_3), 129.8 (*p*-C_6H_3), 127.8 (superimposed by solvent signal of C_6D_6, *p*-C_6H_3), 122.8 (*m*-C_{Trip}), 121.9 (*m*-C_{Trip}), 87.7 (C_5H_5), 34.6 (*o*/*p*-*C*HMe₂), 34.5 (*o*/*p*-*C*HMe₂), 30.9 (*o*/*p*-*C*HMe₂), 30.7 (*o*/*p*-*C*HMe₂), 25.3 (*o*/*p*-CHMe_2), 24.9 (*o*/*p*-CHMe_2), 23.6 (*o*/*p*-CHMe_2), 23.4 (*o*/*p*-CHMe_2), 23.3 (*o*/*p*-CHMe_2). **^{119}Sn NMR** (93.28 MHz, C_6D_6 + 1,2-Difluorobenzene): δ (ppm) 1484 (d, $^1J_{119Sn-H}$ = 550 Hz, Ti*Sn*H), 1789 (s, Ti*Sn*). **^{11}B NMR** (96.29 MHz, C_6D_6 + 1,2-Difluorobenzene): δ (ppm) −25 (d, $^1J_{11B-H}$ = 91 Hz, *B*H). ν Sn–H 1740 cm^{-1}. Anal. calcd. for $C_{100}H_{110}BF_{15}Sn_2Ti$: C 63.45, H 5.86; found: C 63.18, H 5.85.

2. Crystal structure analysis

X-ray crystal structure analysis: X-ray data were collected with a Bruker Smart APEX II diffractometer with graphite monochromated Mo Kα radiation or a Bruker APEX II Duo diffractometer with a Mo IµS microfocus tube. The programs used were Bruker´s APEX2 v2011.8-0 including SAINT for data reduction and SHELXS for structure solution and SADABS for multiscan absorption correction, as well as WinGX suite of programs v1.70.01 including SHELXL for structure refinement.[2]

Table S1. Crystal structure refinement table of compounds **4**-**7**

**compound**	**4**	**5**	**6**	**7**
Empirical formula	$C_{68}H_{118}Sn_2TiO$	$C_{89}H_{118}Sn_2Zr$	$C_{82}H_{110}HfSn_2$	$C_{60.5}H_{67}B_{0.5}F_{10}SnTi_{0.5}$
M_r [g mol^{-1}]	1453.08	1516.43	1511.56	1132.18
T [K]	100(2)	100(2)	100(2)	100(2)
λ [Å]	0.71073	0.71073	0.71073	0.71073
Crystal system	triclinic	triclinic	triclinic	triclinic
Space group	$P\bar{1}$	$P\bar{1}$	$P\bar{1}$	$P\bar{1}$
Z	2	2	2	2
a [Å]	13.6476(6)	13.5703(3)	13.5900(6)	13.5899(4)
b [Å]	14.4031(6)	14.5485(3)	14.5206(6)	19.5075(6)
c [Å]	20.5226(9)	21.1413(4)	20.8846(9)	21.7717(7)
α [°]	107.082(2)	72.0210(10)	72.342(2)	70.009(2)
β [°]	91.018(3)	89.5100(10)	89.363(3)	81.886(2)
γ [°]	98.937(2)	79.4750(10)	80.132(3)	86.383(2)
V [Å^3]	3800.7(3)	3898.16(14)	3865.3(3)	5369.0(3)
D_{calc} [g cm^{-3}]	1.270	1.292	1.299	1.401
μ [mm^{-1}]	0.799	0.810	2.020	0.618
F(000)	1528	1584	1548	2334
Crystal size [mm]	0.15x0.14x0.11	0.15x0.13x0.11	0.2×0.16×0.12	0.18×0.16×0.15
θ range[°]	1.778-25.027	1.877-27.541	1.522-29.505	1.892-25.390
Limiting indices	-16≤*h*≤16	-17≤*h*≤17	-18≤*h*≤18	-16≤*h*≤16
	-17≤*k*≤17	-18≤*k*≤18	-20≤k≤20	-23≤*k*≤23
	-22≤*l*≤24	-27≤*l*≤27	-28≤l≤28	-26≤*l*≤26
Collected refl.	68105	70295	109120	89160
Independent refl.	13283	17865	21288	19617
R_{int}	0.0563	0.0566	0.0540	0.0551
completeness	98.9	99.4	98.8	99.3
Absorption corr.	multi-scan	multi-scan	multi-scan	multi-scan
Trans. (max., min.)	0.7454, 0.6304	0.7456, 0.6773	0.7458, 0.6083	0.7452, 0.5802
Parameter/Restraints	783/74	880/2	778/2	1215/1

R_1, ωR_2 [$I > 2\sigma(I)$]	0.0701, 0.1664	0.0505, 0.0991	0.0412, 0.0958	0.0458, 0.1119
R_1, ωR_2 (all data)	0.0986, 0.1802	0.0871, 0.1108	0.0599, 0.1045	0.0758, 0.1245
GooF on F^2	1.090	1.020	1.045	1.020
$\Delta\rho_{max,min}$ [e·Å^{-3}]	2.127/-1.872	2.469/-1.504	3.745/-1.589	1.157/-0.805
CCDC	1944501	1944498	1944500	1944499

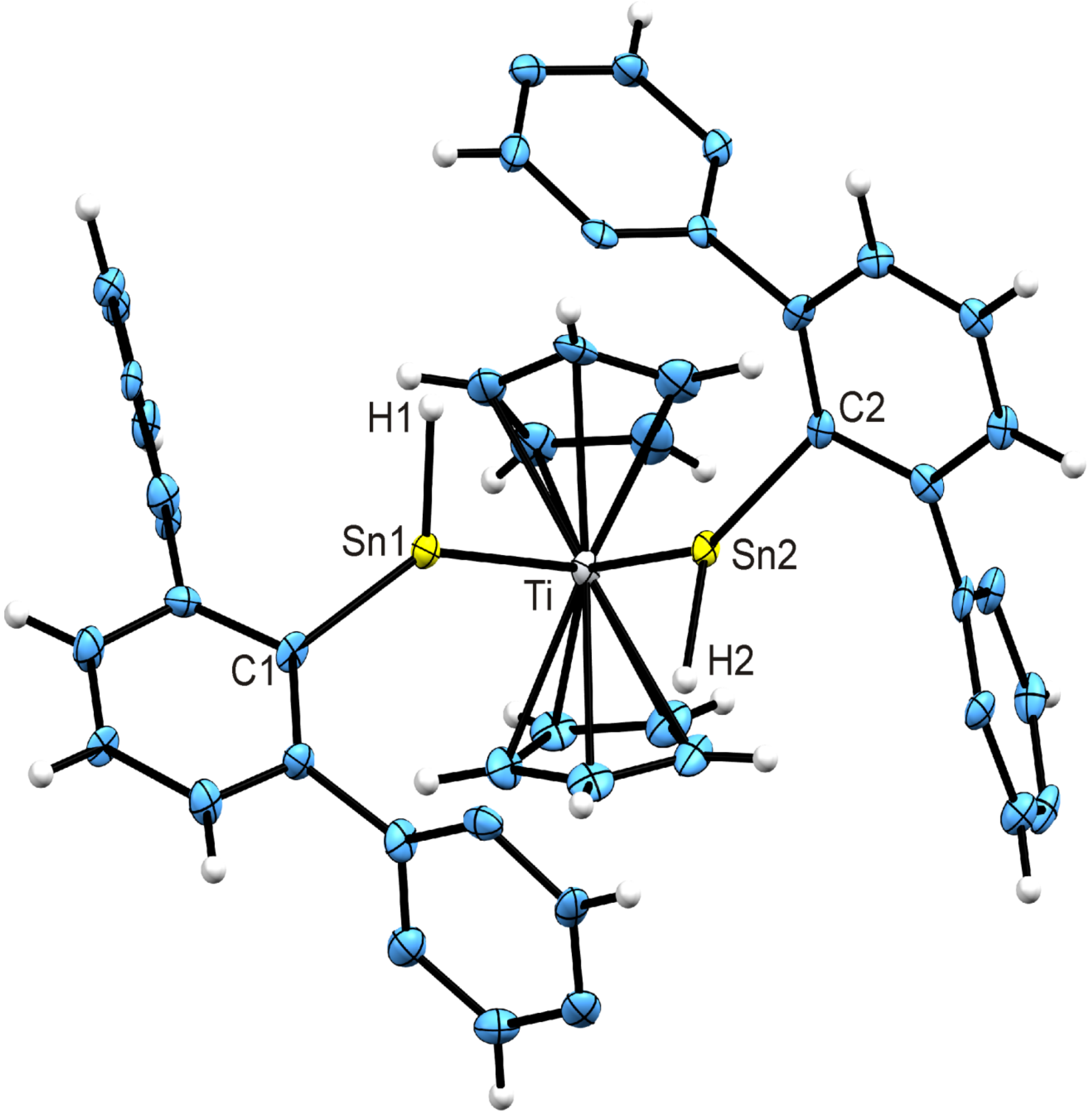

Figure S1. ORTEP of **4**. Iso-Pr groups were omitted for the sake of clarity. Hydrogen atoms connected to

the tin atom were found other hydrogen atoms were placed in idealized positions. Ellipsoids at 50% probability.

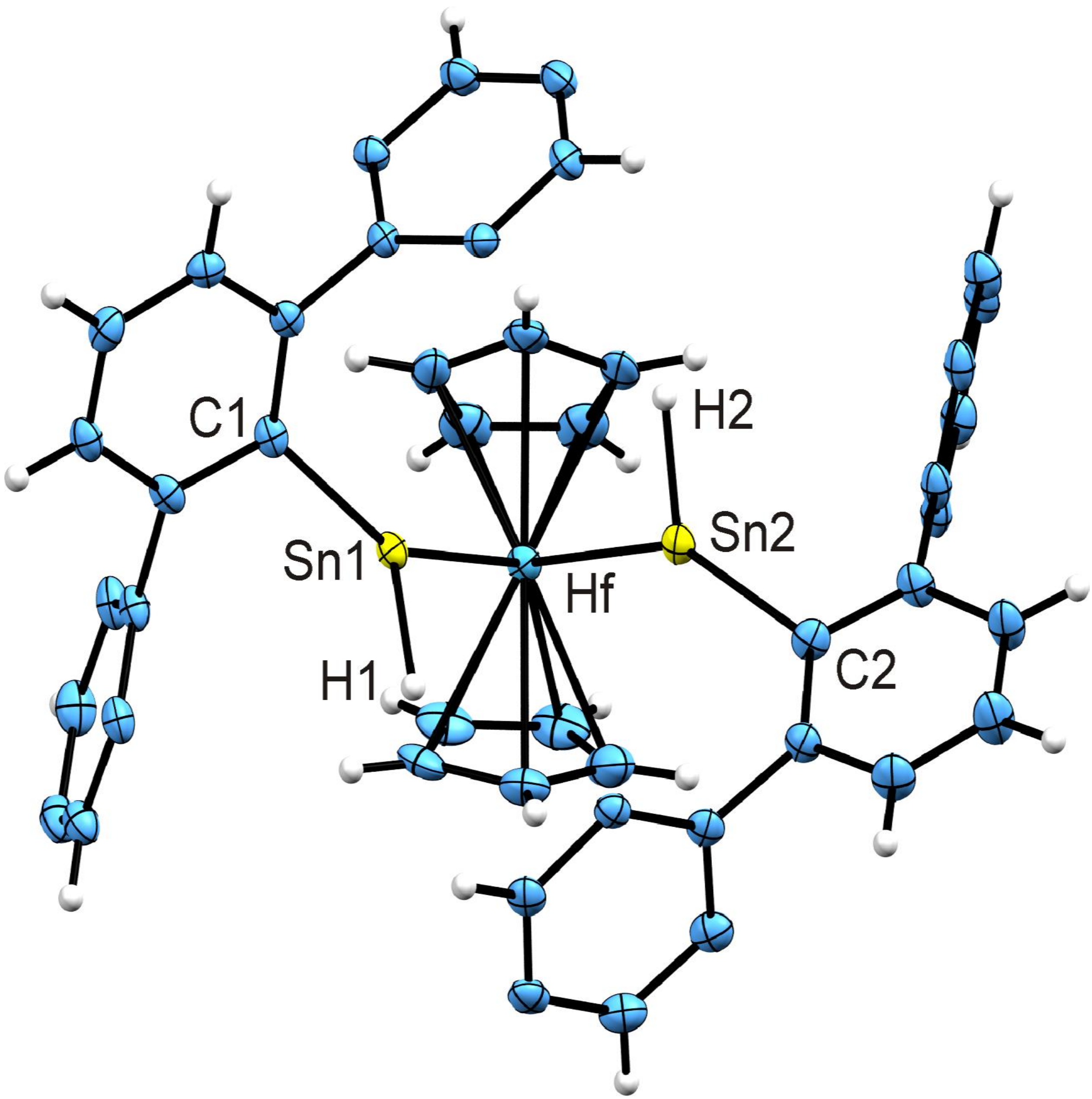

Figure S2. ORTEP of **6**. Iso-Pr groups were omitted for the sake of clarity. Hydrogen atoms connected to the tin atom were found other hydrogen atoms were placed in idealized positions. Ellipsoids at 50% probability.

3. NMR data

3.1 NMR spectra of compound **2**

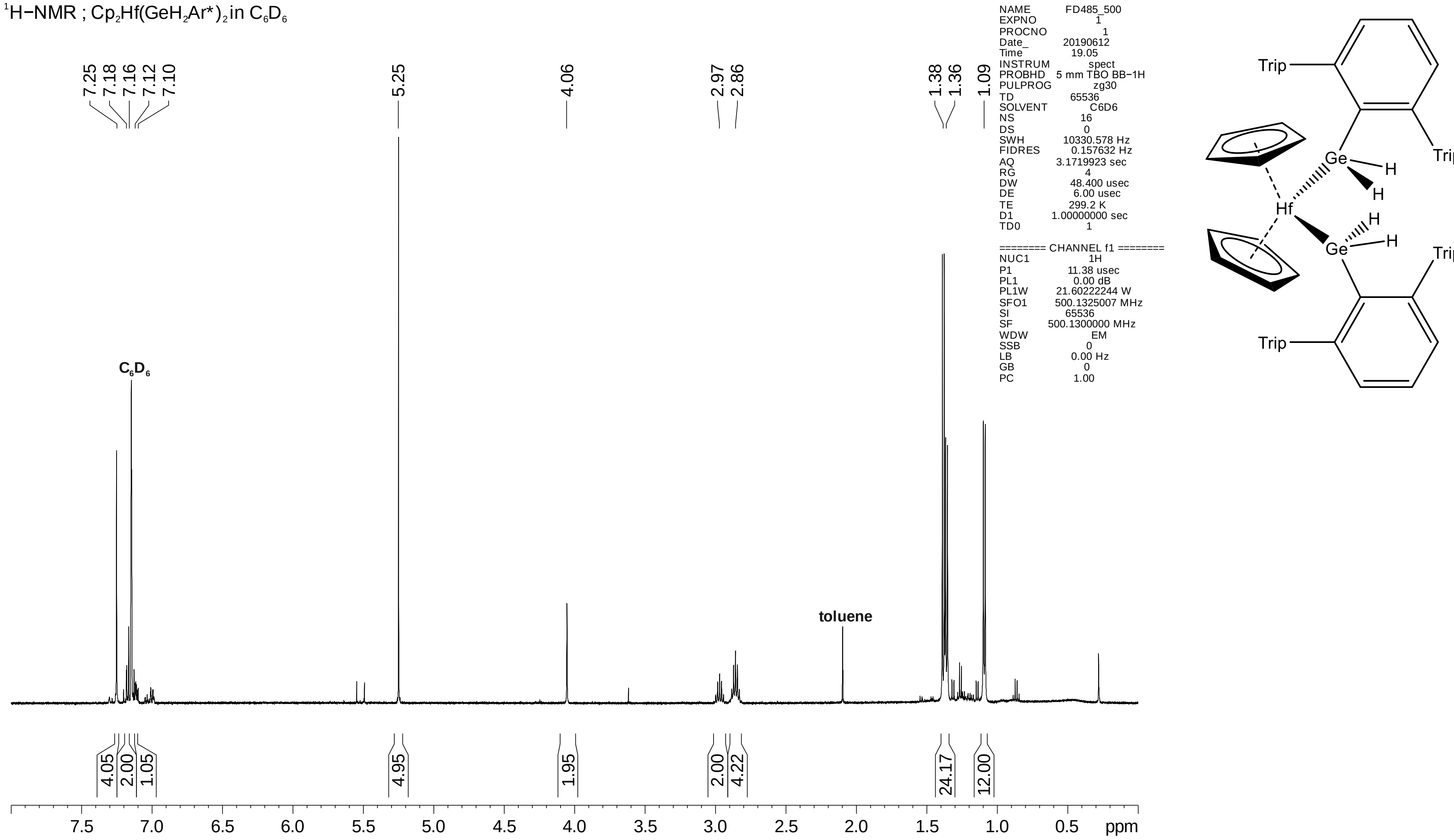

Figure S3. ^{1}H NMR spectrum of compound **2**.

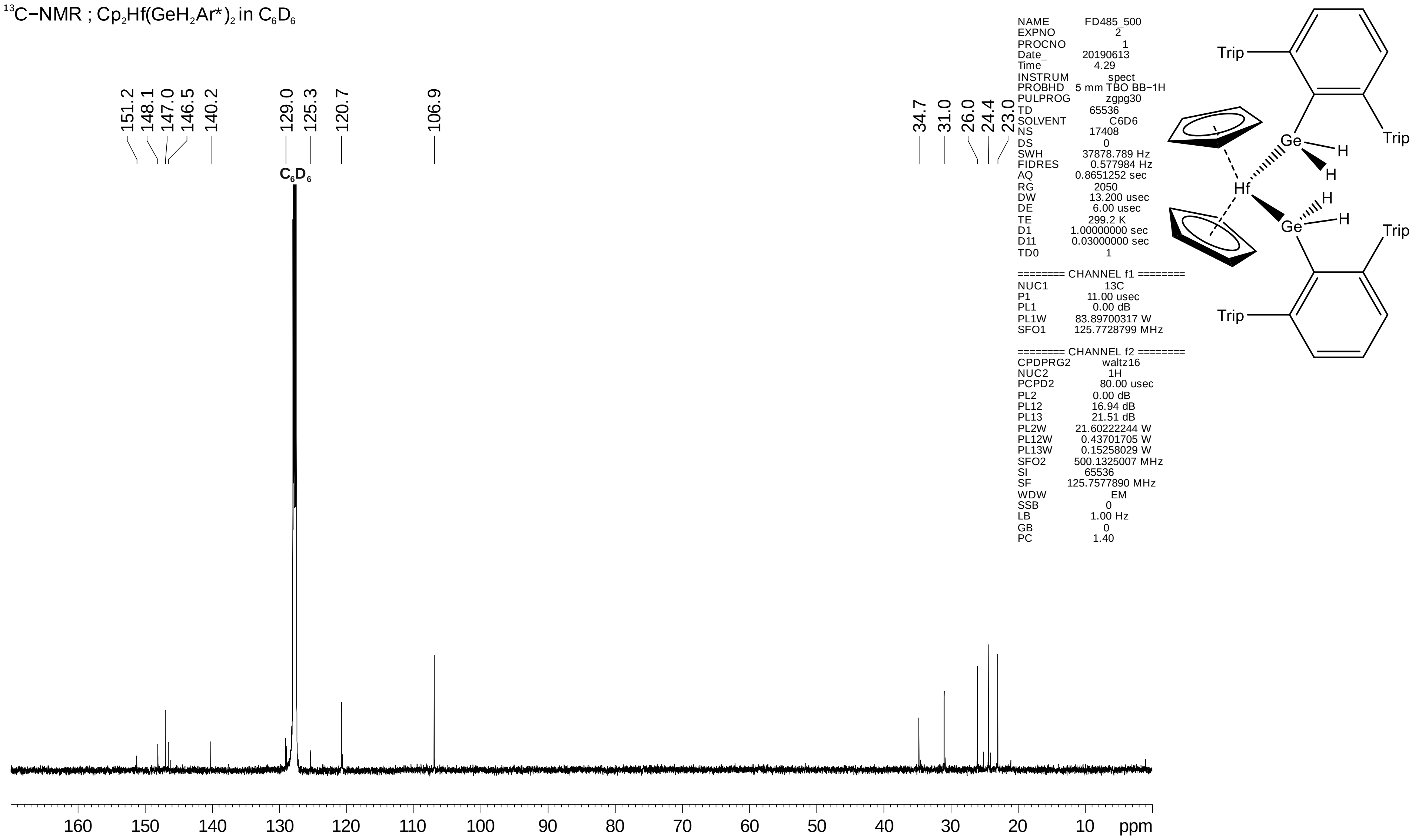

Figure S4. $^{13}C\{^{1}H\}$ NMR spectrum of compound **2**.

3.2 NMR spectra of compound **4**

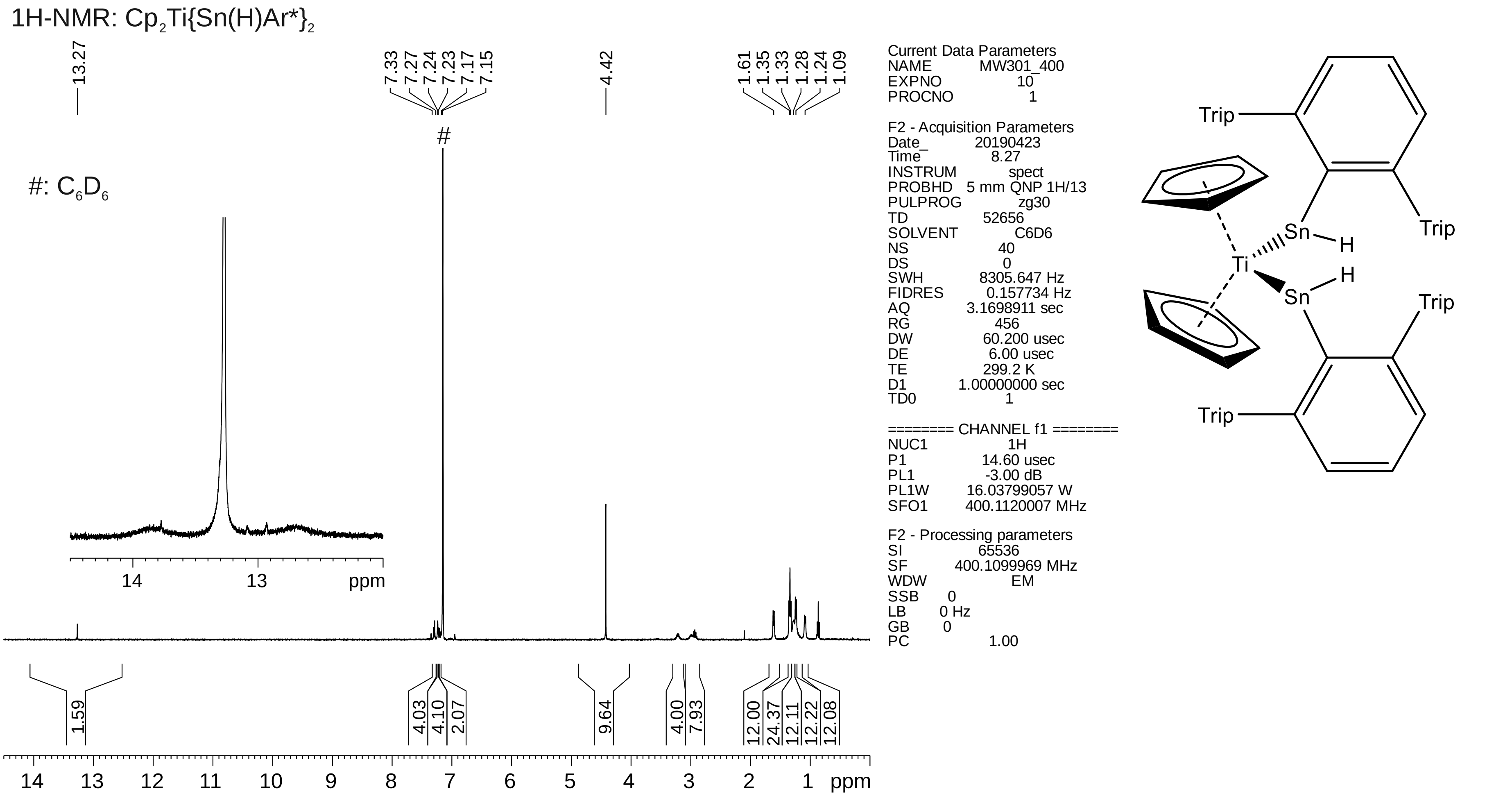

Figure S5. ^{1}H NMR spectrum of compound **4**.

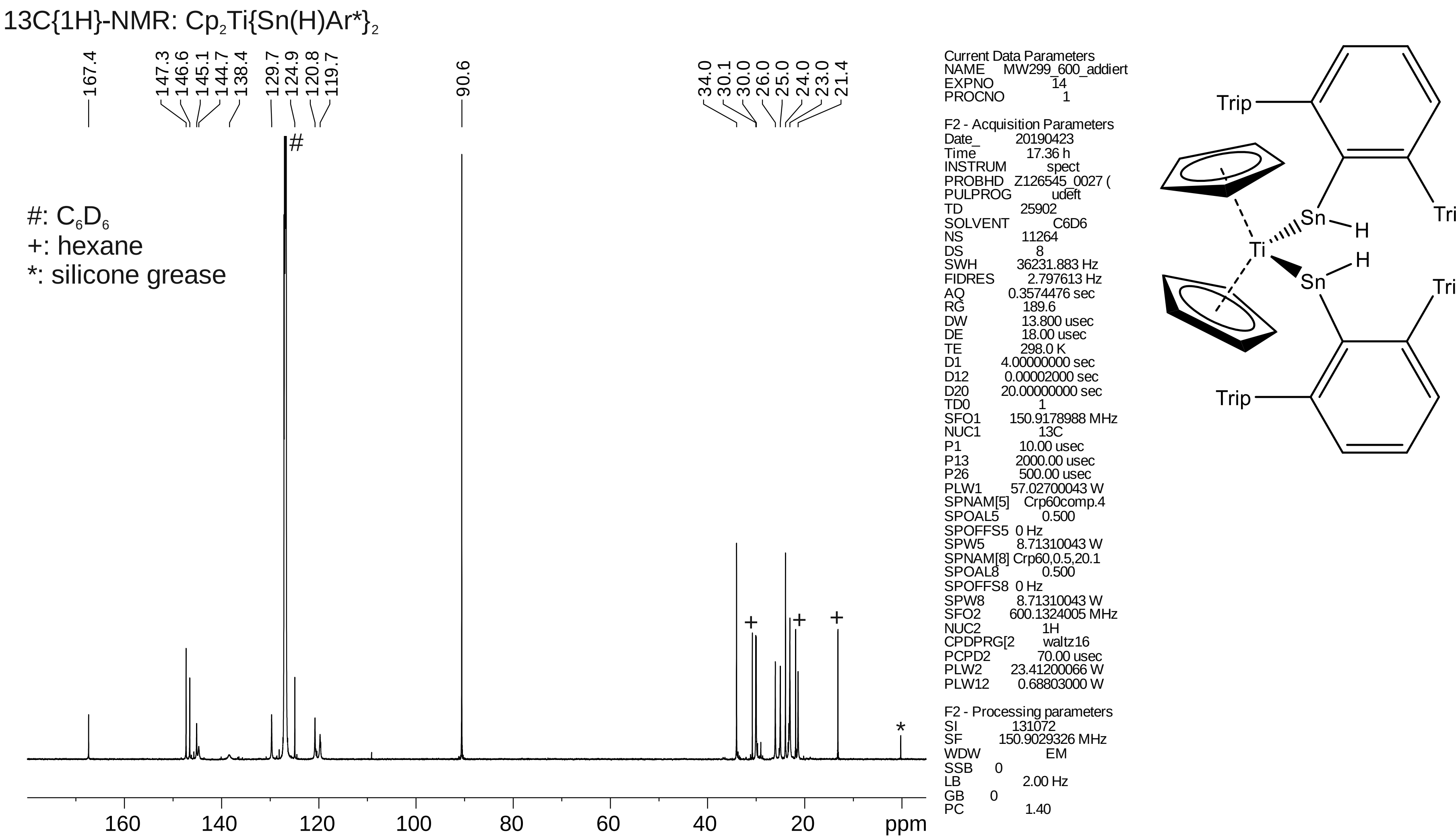

Figure S6. $^{13}C\{^{1}H\}$ NMR spectrum of compound **4**.

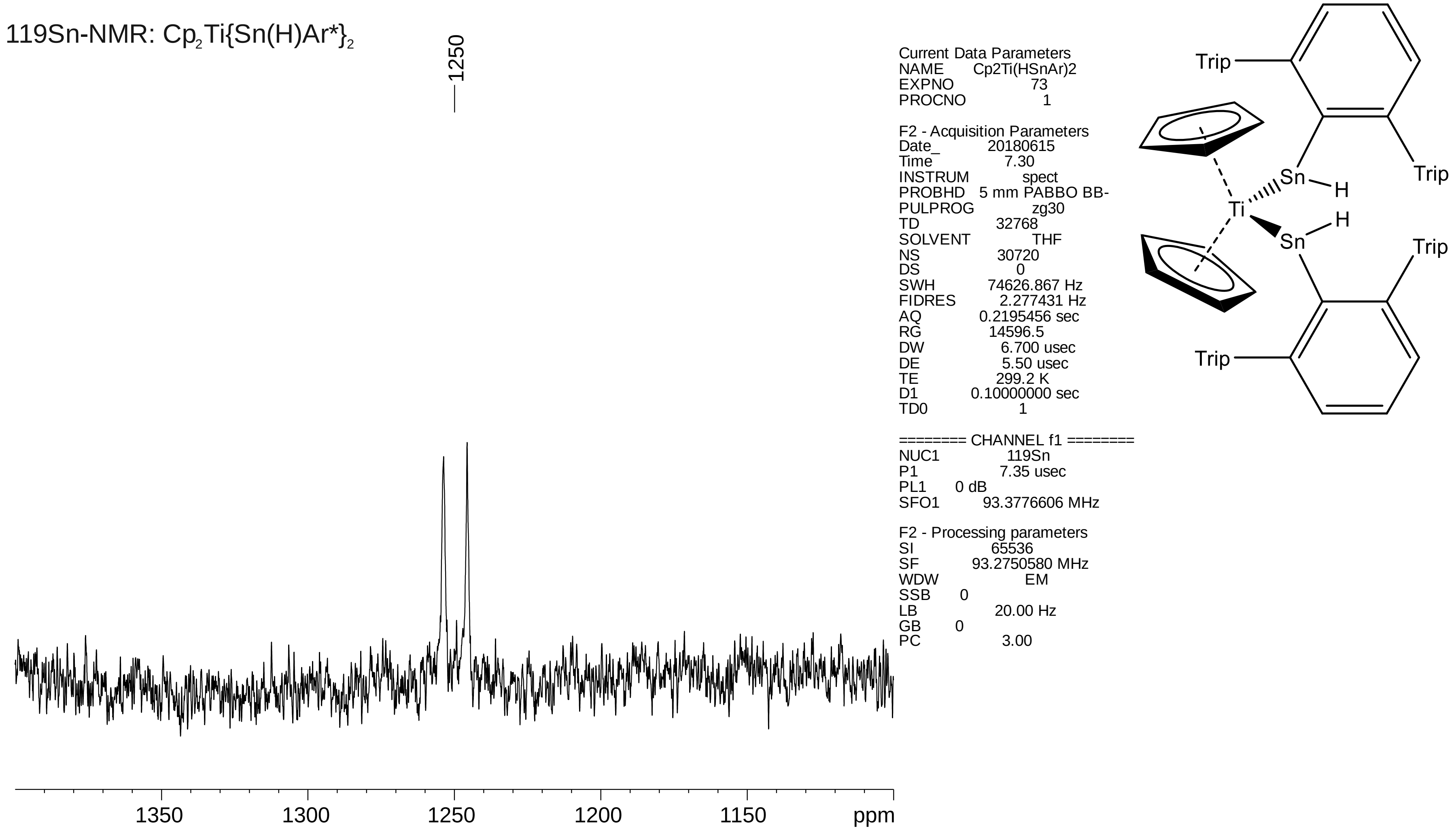

Figure S7. ^{119}Sn NMR spectrum of compound **4**.

3.3 NMR spectra of compound **5**

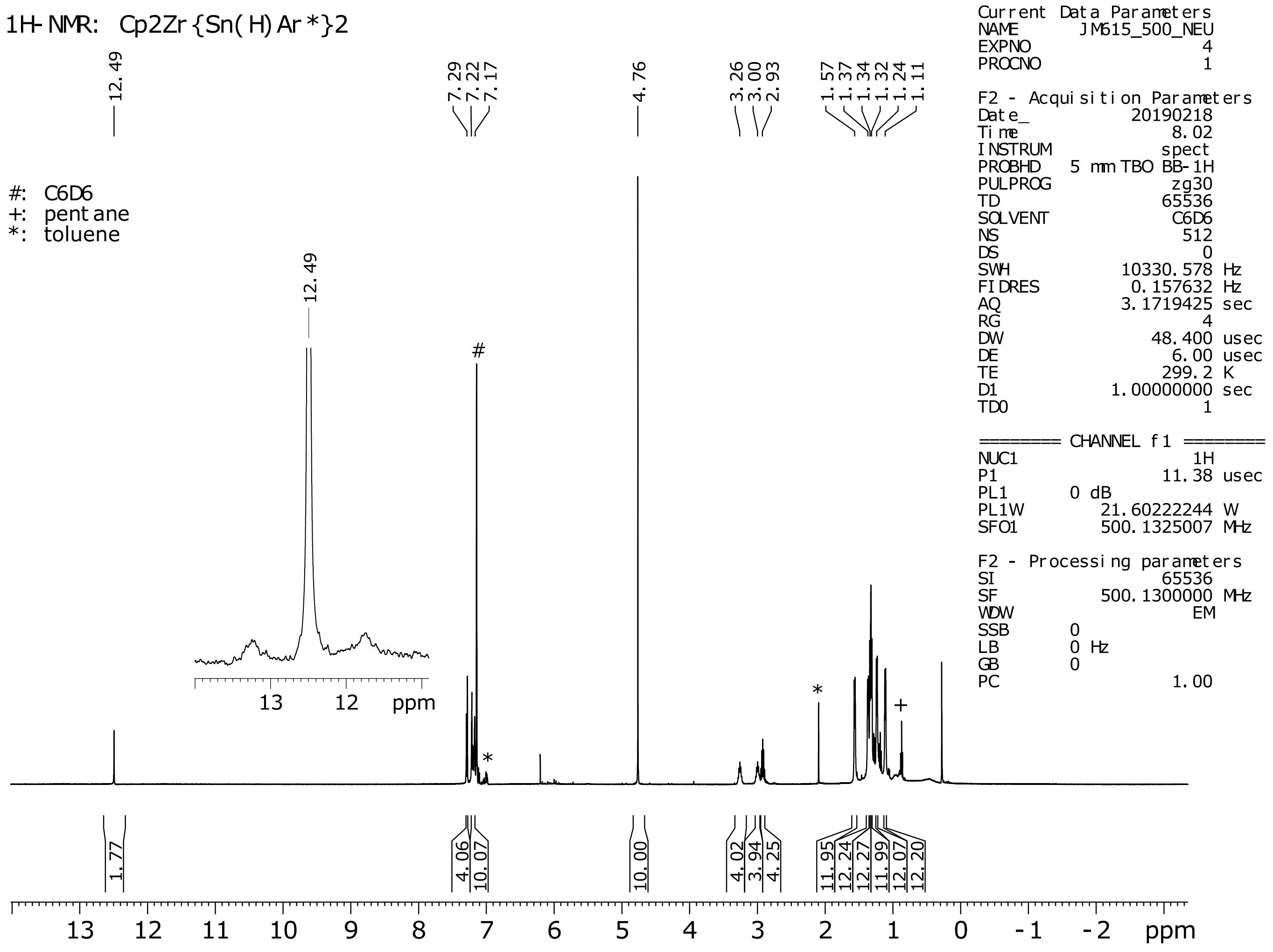

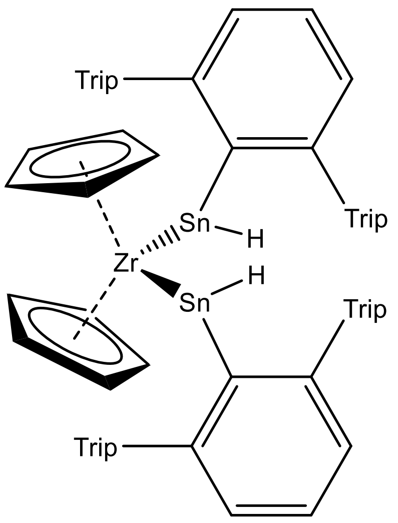

Figure S8. ^{1}H NMR spectrum of compound **5**.

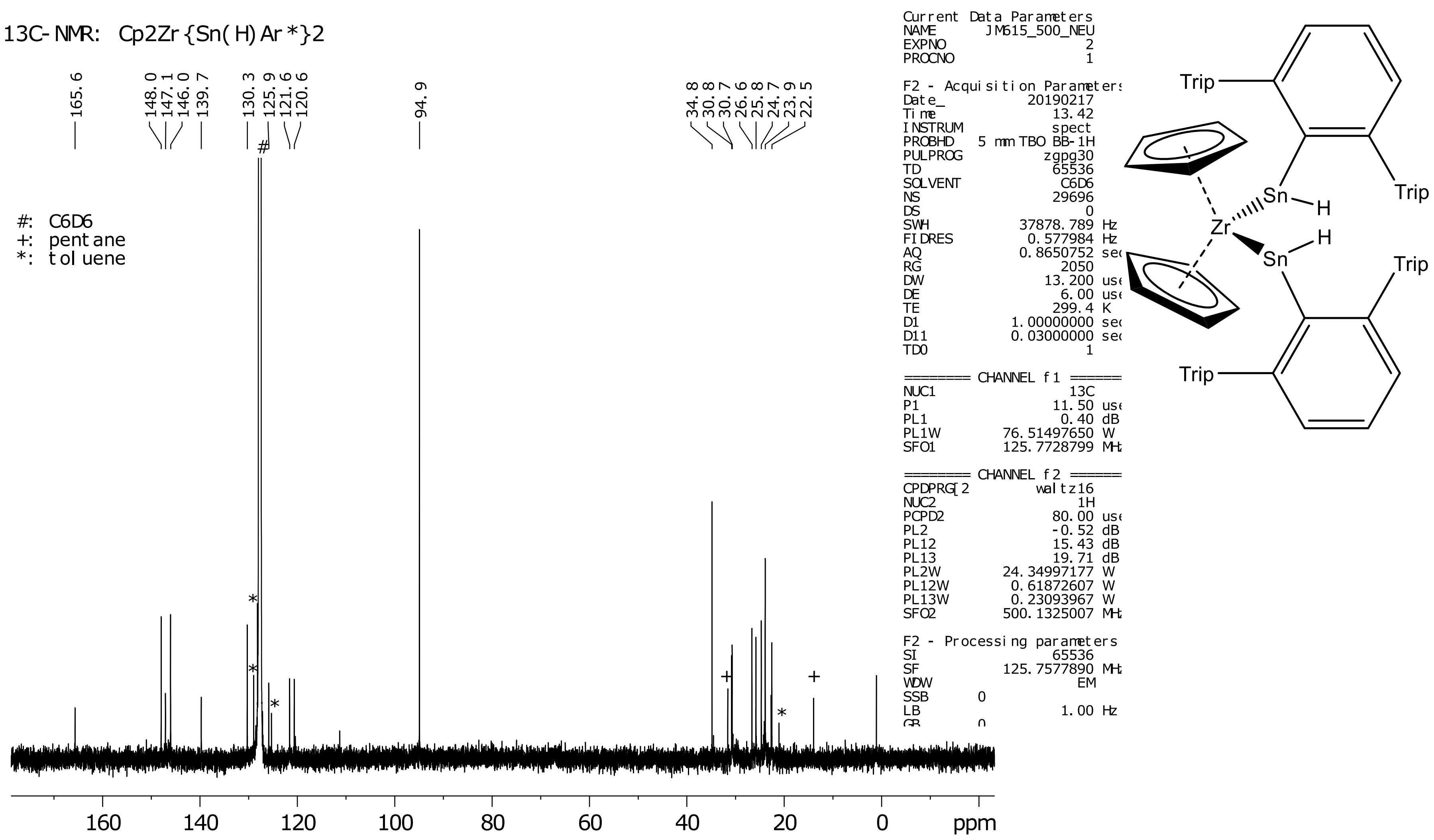

Figure S9. $^{13}C\{^{1}H\}$ NMR spectrum of compound **5**.

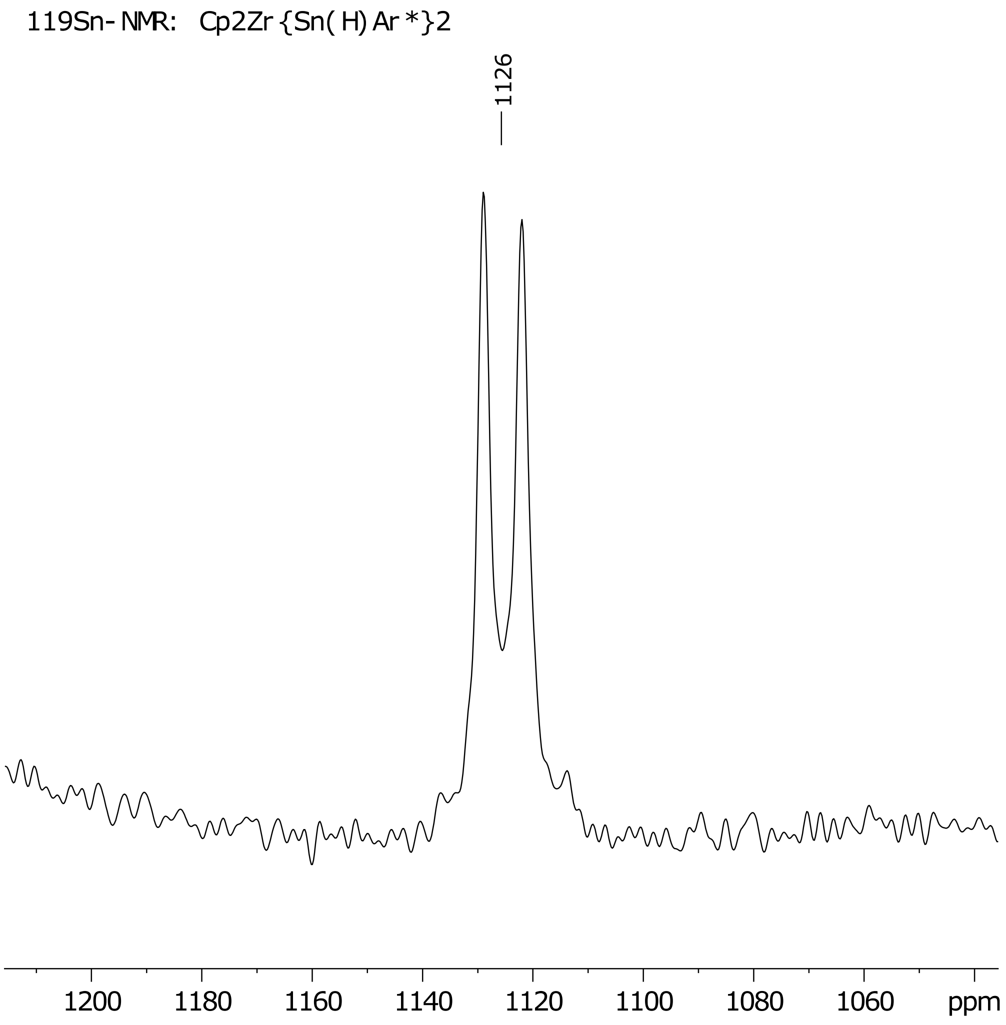


```
Current Data Parameters
NAME      JM598_300_Nachtmessung_FI
EXPNO                    15
PROCNO                    4

F2 - Acquisition Parameters
Date_              20181211
Time                  14.29 h
INSTRUM               spect
PROBHD     Z104275_0338 (
PULPROG               zg30
TD                      882
SOLVENT                C6D6
NS                  1121280
DS                        1
SWH               89285.711 Hz
FIDRES           101.230972 Hz
AQ                0.0049392 sec
RG                   181.04
DW                    5.600 usec
DE                     6.50 usec
TE                    298.0 K
D1               0.02000000 sec
TD0                       1
SFO1            112.0546780 MHz
NUC1                  119Sn
P1                    12.10 usec
PLW1            12.00000000 W

F2 - Processing parameters
SI                     4096
SF              111.9203740 MHz
WDW                      EM
SSB       0
LB        0 Hz
GB        0
PC                     1.40
```

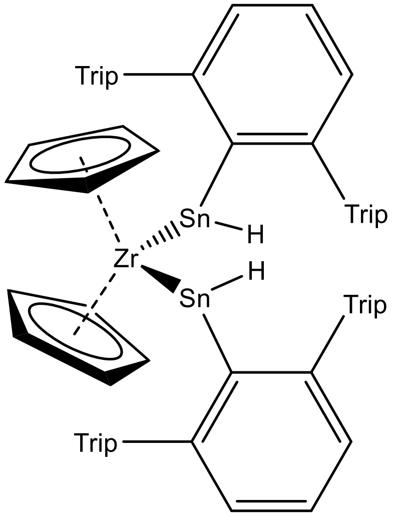

Figure S10. ^{119}Sn NMR spectrum of compound **5**.

3.4 NMR spectra of compound **6**

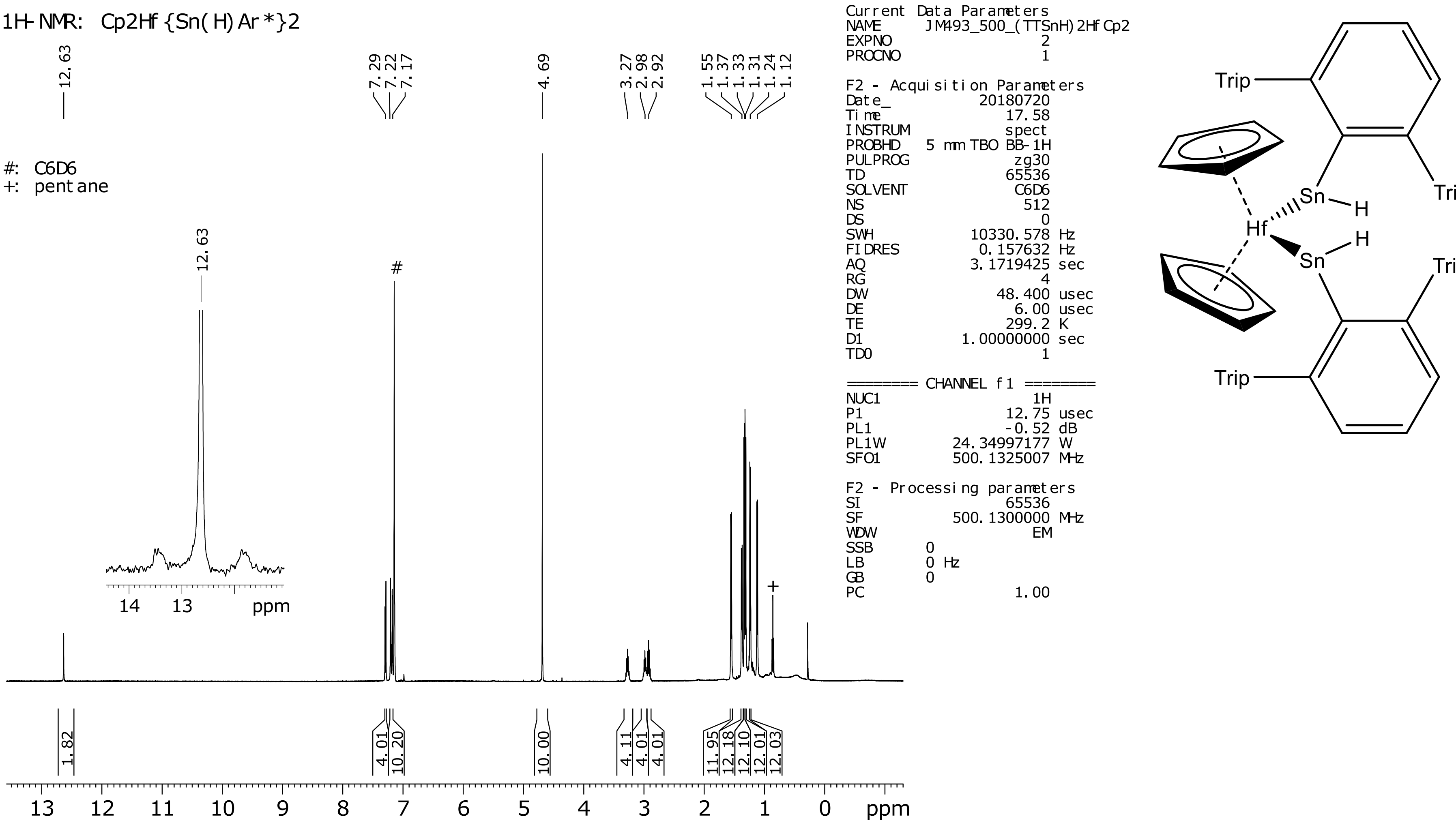

Figure S11. ^{1}H NMR spectrum of compound **6**.

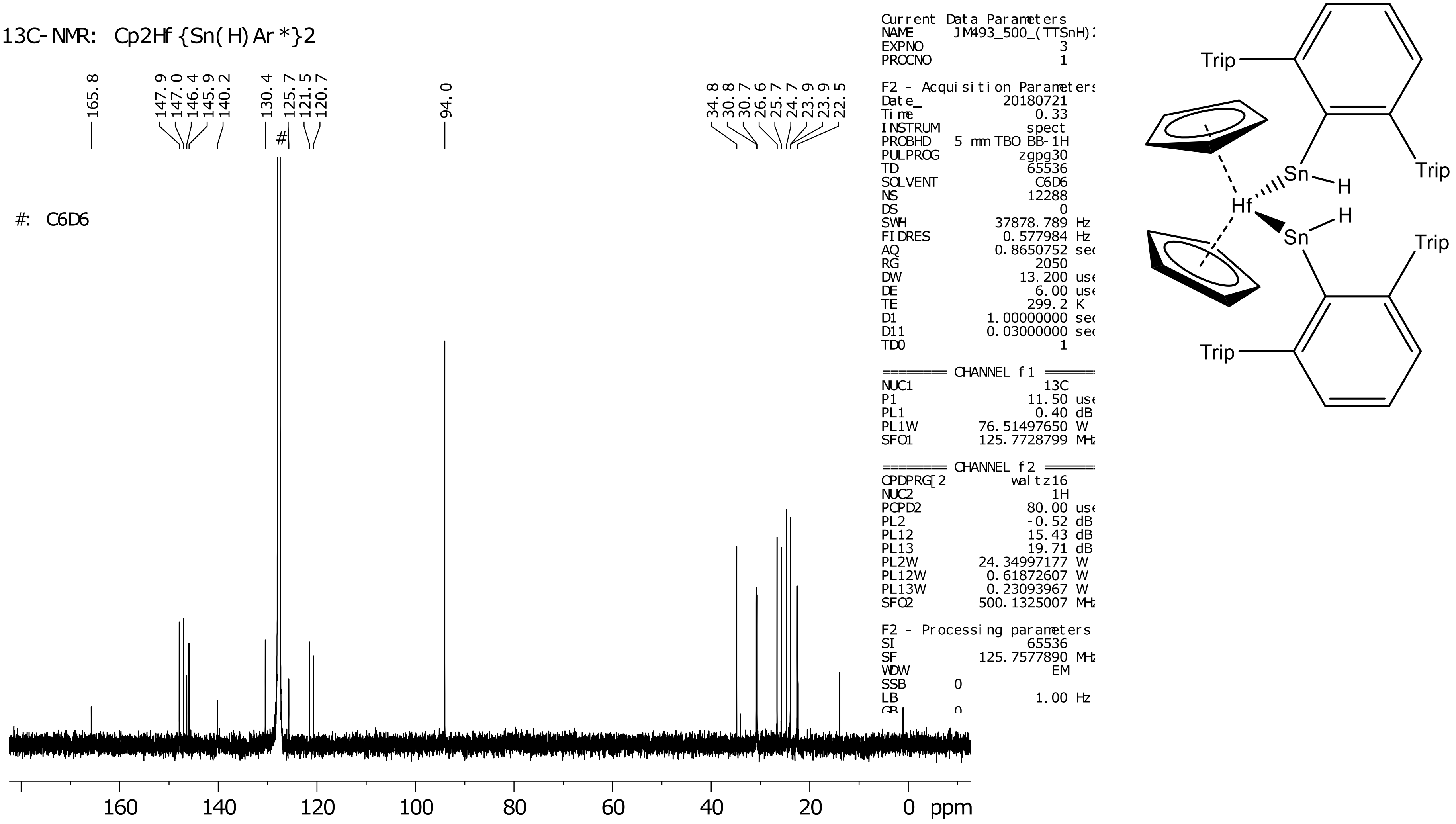

Figure S12. $^{13}C\{^{1}H\}$ NMR spectrum of compound **6**.

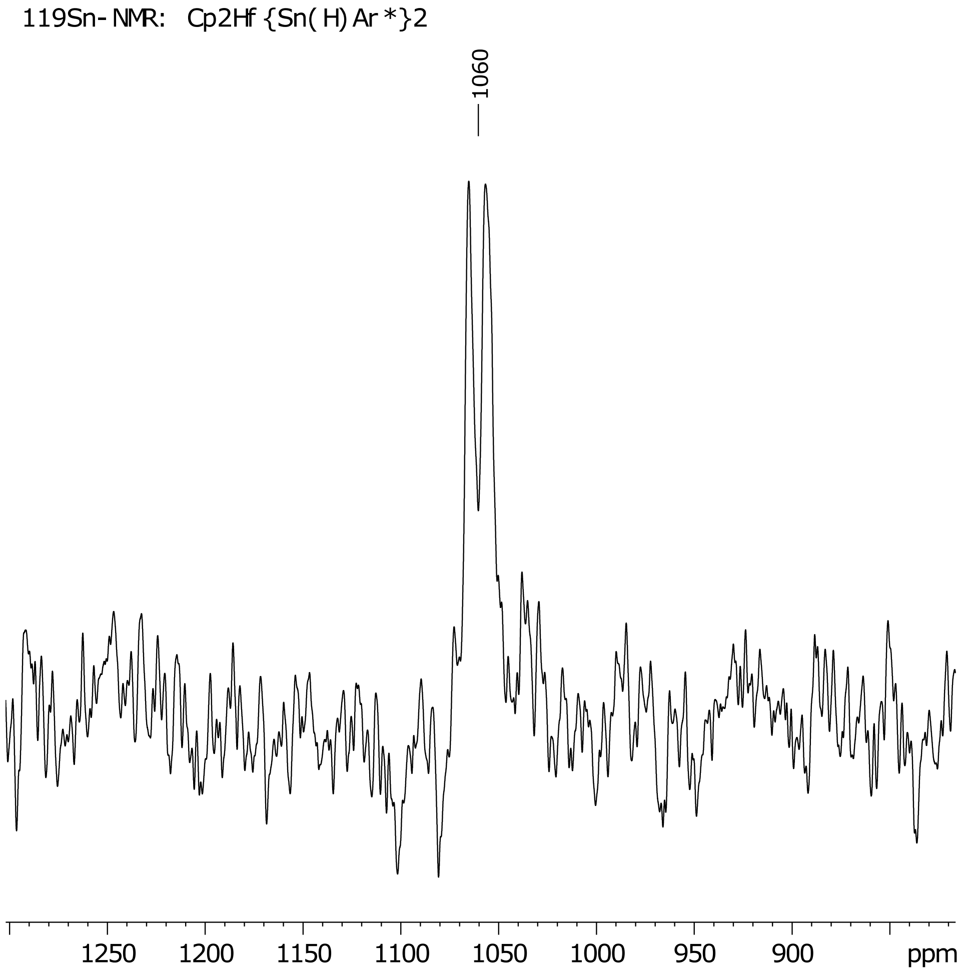


```
Current Data Parameters
NAME        JM493_250_Nacht
EXPNO                    12
PROCNO                    1

F2 - Acquisition Parameters
Date_              20180710
Time                   6.04
INSTRUM               spect
PROBHD   5 mm PABBO BB-
PULPROG                zg30
TD                    32768
SOLVENT                C6D6
NS                   102400
DS                        0
SWH              74626.867 Hz
FIDRES            2.277431 Hz
AQ               0.2195456 sec
RG                  14596.5
DW                    6.700 usec
DE                     5.50 usec
TE                    299.2 K
D1               0.10000000 sec
TD0                       1

======== CHANNEL f1 ========
NUC1                  119Sn
P1                     7.35 usec
PL1         0 dB
SFO1           93.3869881 MHz

F2 - Processing parameters
SI                    65536
SF             93.2750580 MHz
WDW                      EM
SSB         0
LB                   150.00 Hz
GB          0
PC                     3.00
```

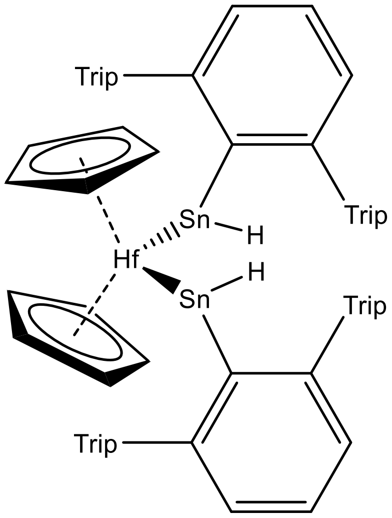

Figure S13. ^{119}Sn NMR spectrum of compound **6**.

3.5 NMR spectra of compound **7**

1H-NMR: [Cp_2Ti{SnAr*}{Sn(H)Ar*}][HB(C_6F_5)$_3$]:

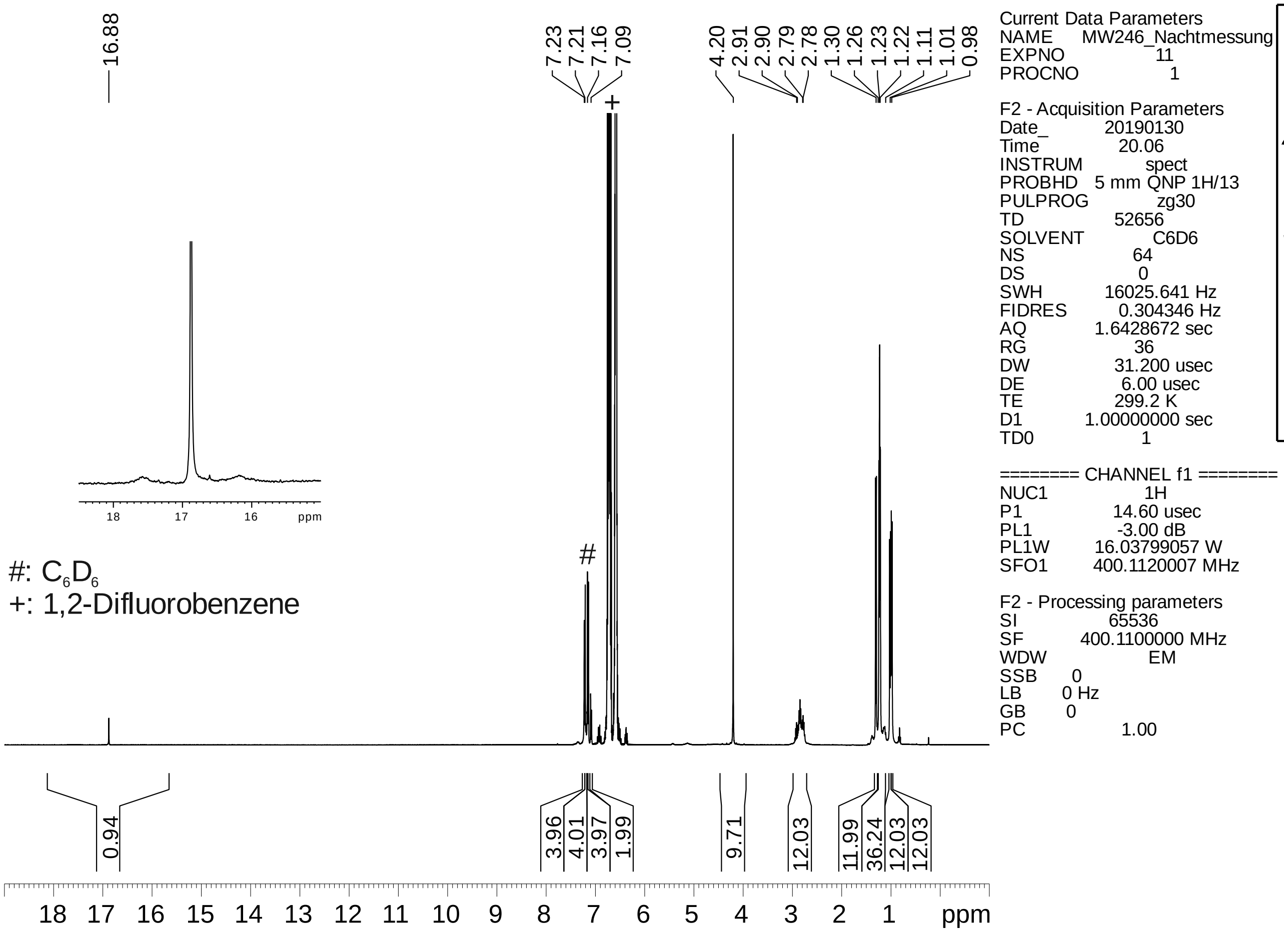

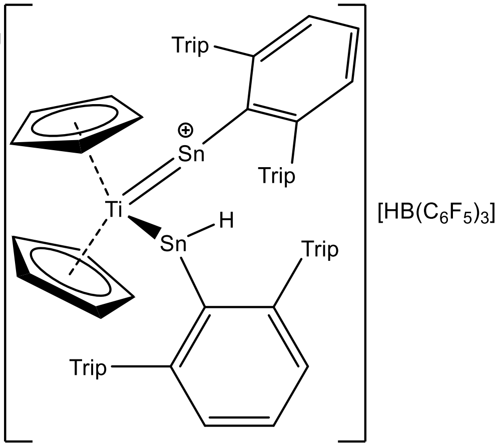

Figure S14. ^{1}H NMR spectrum of compound **7**.

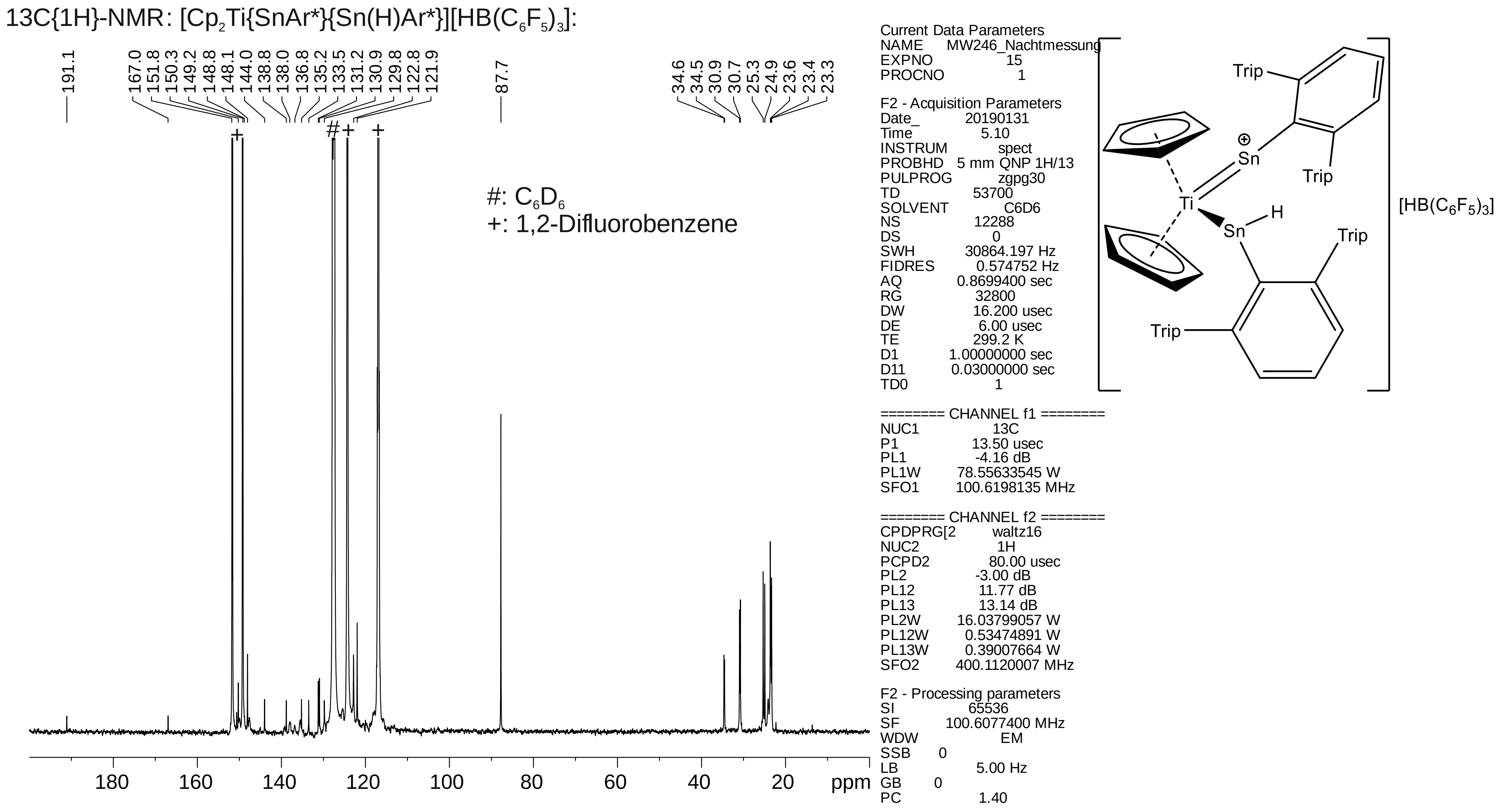

Figure S15. $^{13}C\{^{1}H\}$ NMR spectrum of compound **7**.

119Sn-NMR: $[Cp_2Ti\{SnAr^*\}\{Sn(H)Ar^*\}][HB(C_6F_5)_3]$:

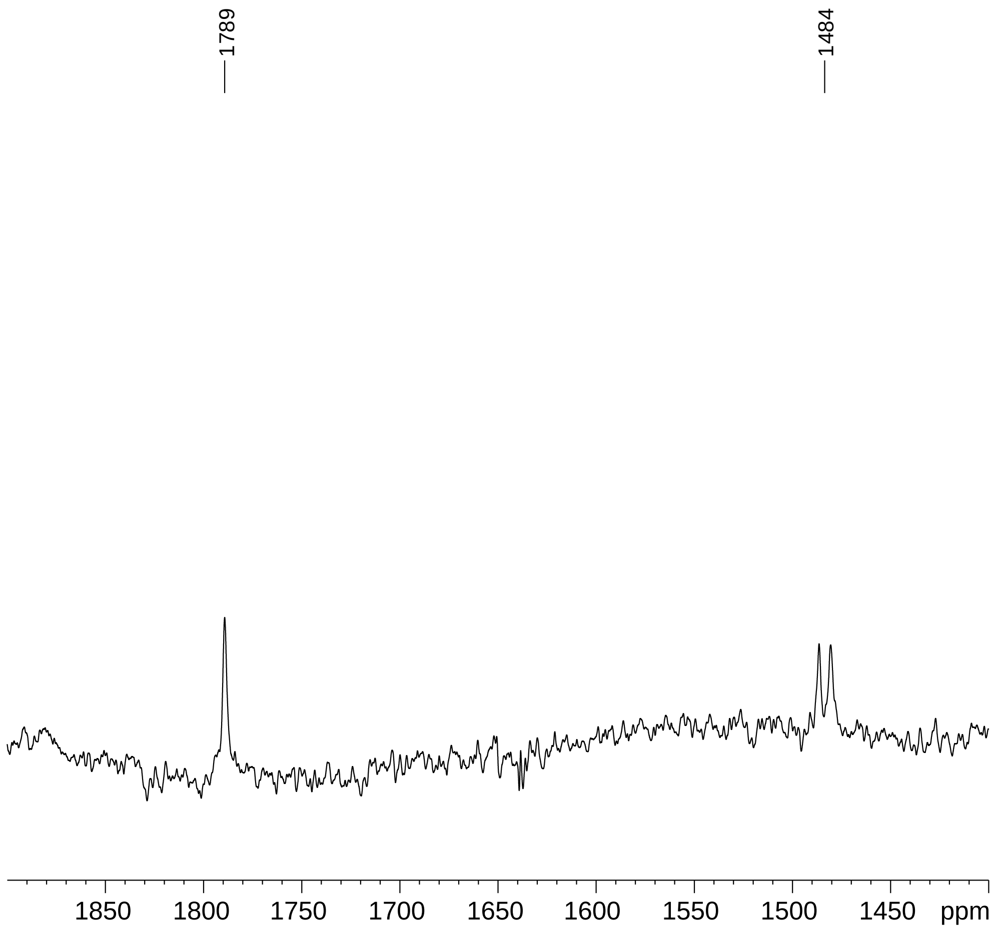

Current Data Parameters
NAME MW161_250
EXPNO 13
PROCNO 1

F2 - Acquisition Parameters
Date_ 20180913
Time 1.50
INSTRUM spect
PROBHD 5 mm PABBO BB-
PULPROG zg30
TD 32768
SOLVENT C6D6
NS 116000
DS 0
SWH 74626.867 Hz
FIDRES 2.277431 Hz
AQ 0.2195456 sec
RG 14596.5
DW 6.700 usec
DE 5.50 usec
TE 299.2 K
D1 0.10000000 sec
TD0 1

======== CHANNEL f1 ========
NUC1 119Sn
P1 7.35 usec
PL1 0 dB
SFO1 93.4289618 MHz

F2 - Processing parameters
SI 65536
SF 93.2750580 MHz
WDW EM
SSB 0
LB 100.00 Hz
GB 0
PC 3.00

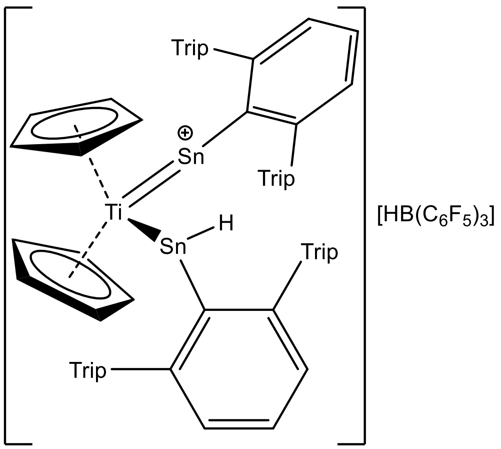

Figure S16. ^{119}Sn NMR spectrum of compound **7**.

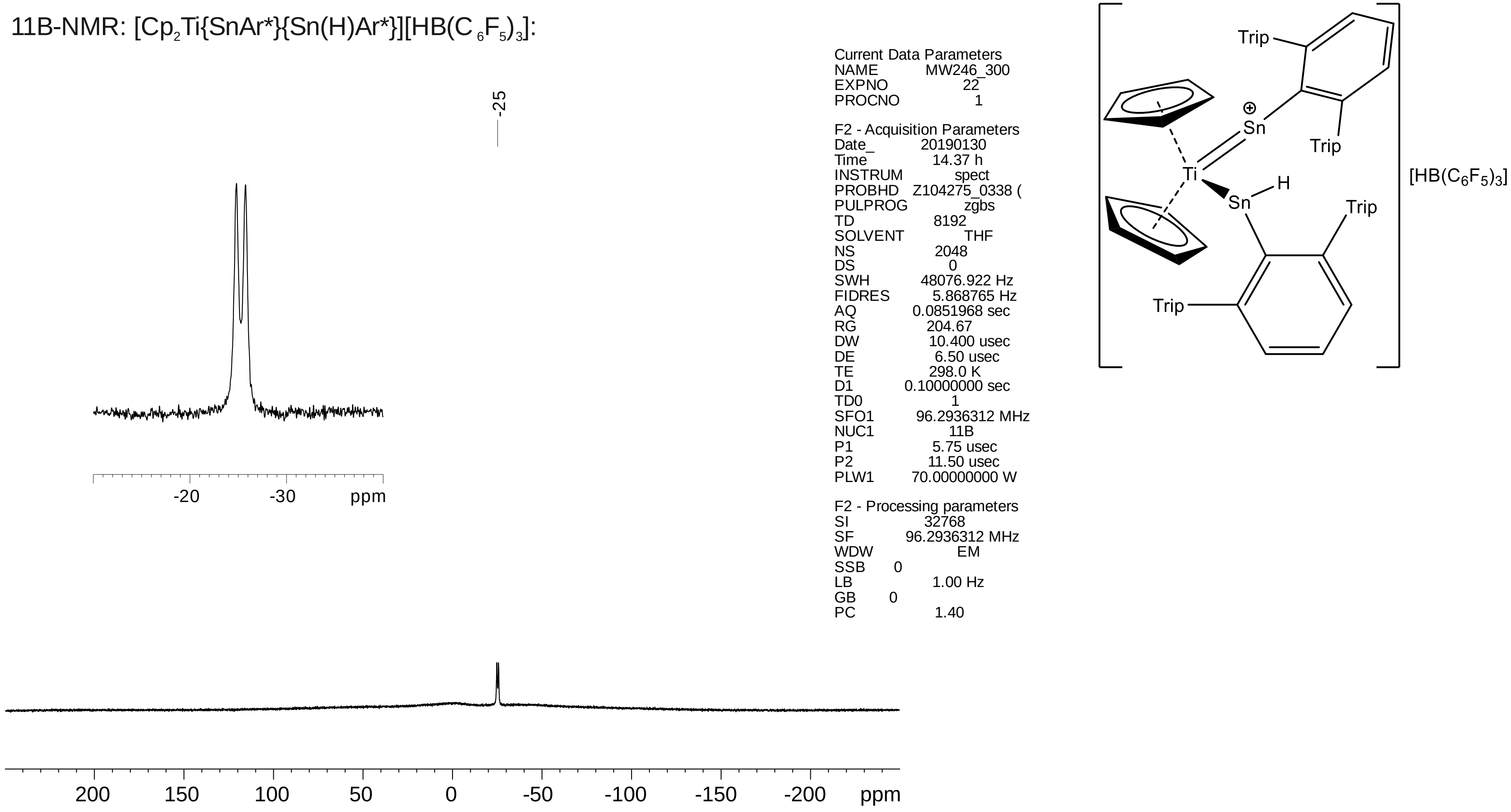

Figure S17. ^{11}B NMR spectrum of compound **7**.

4. IR spectra

[$Cp_2Hf(GeH_2Ar^*)_2$]

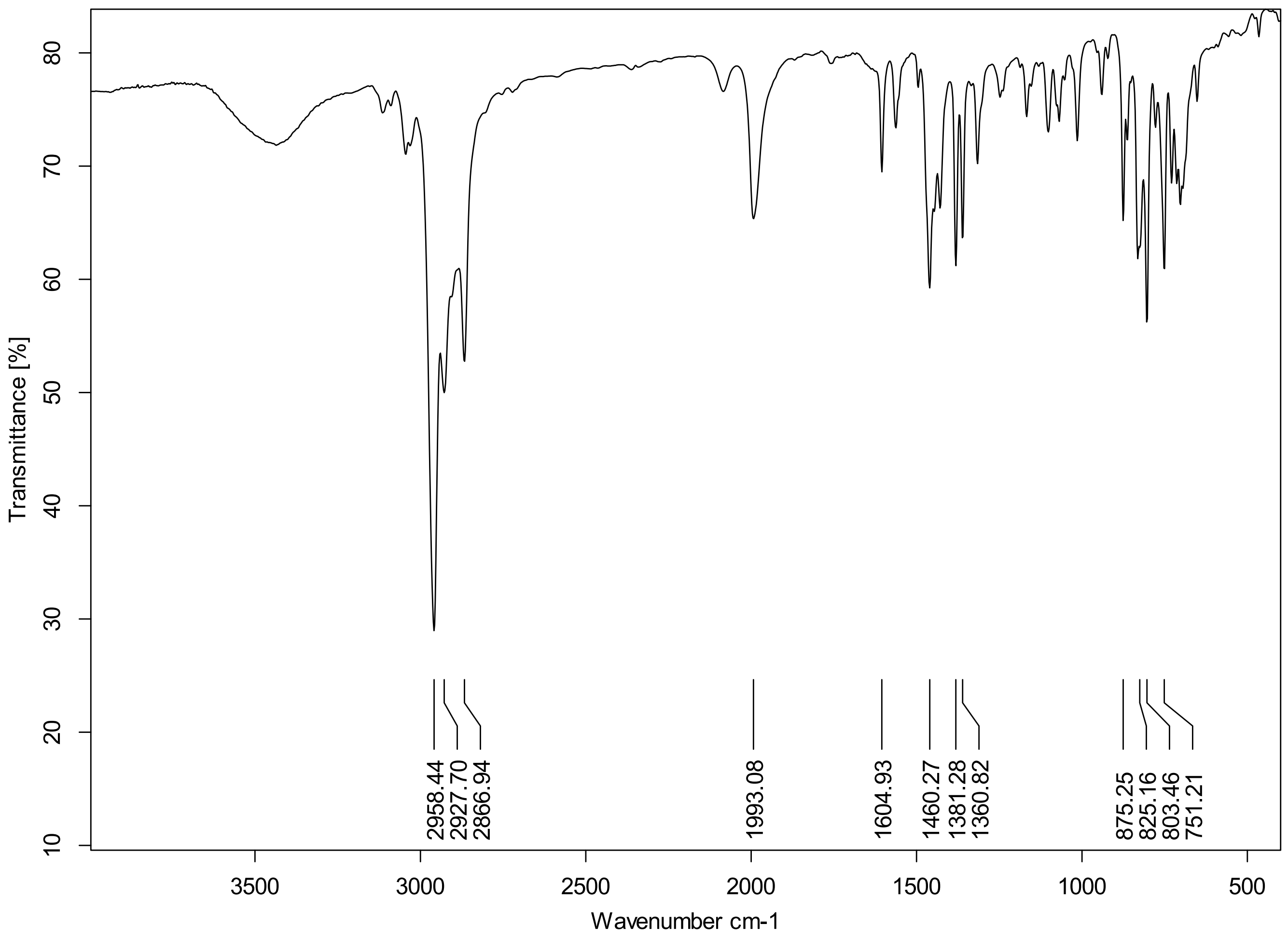

Figure S18. IR spectrum of **2**.

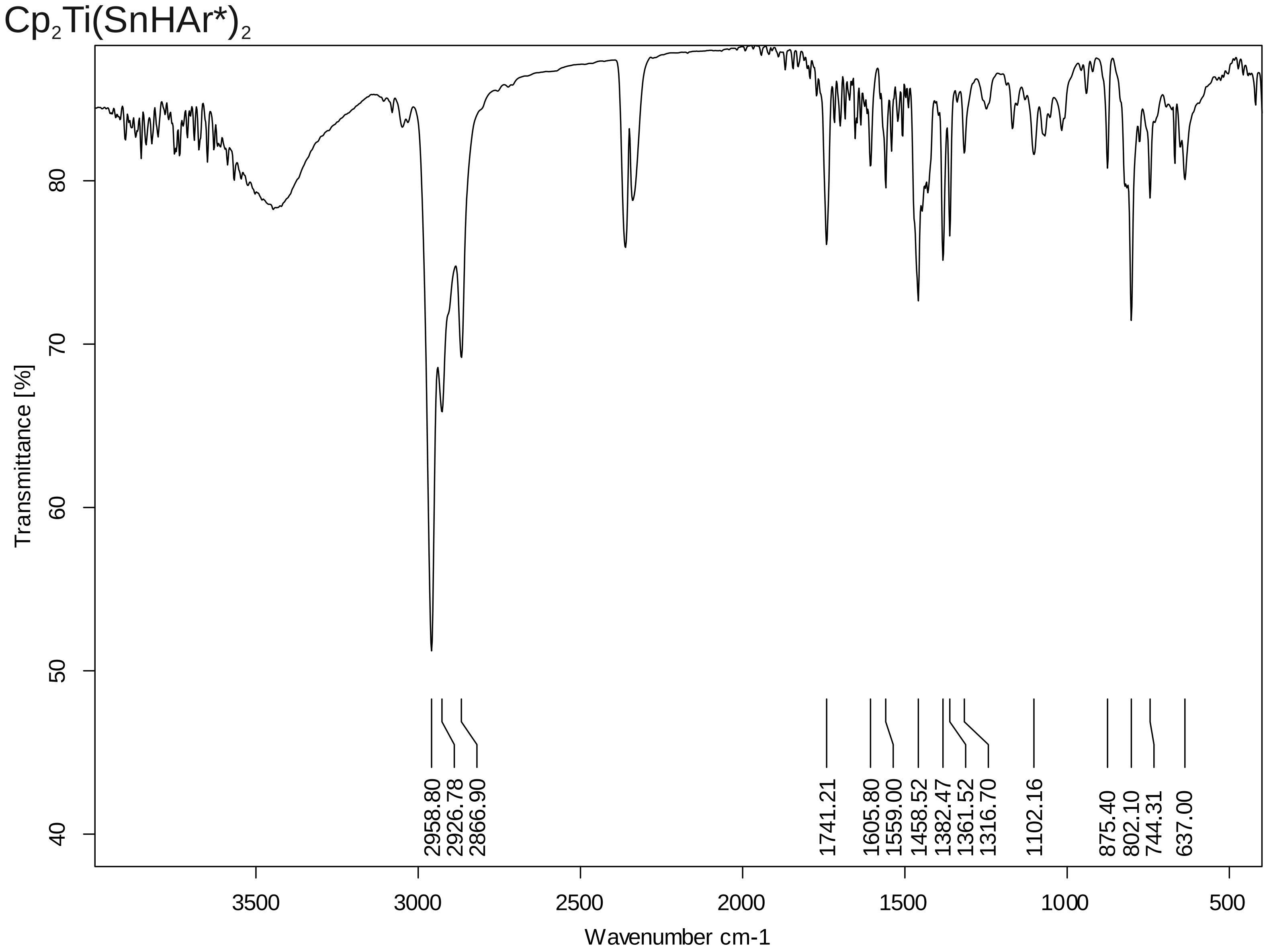

Figure S19. IR spectrum of **4**.

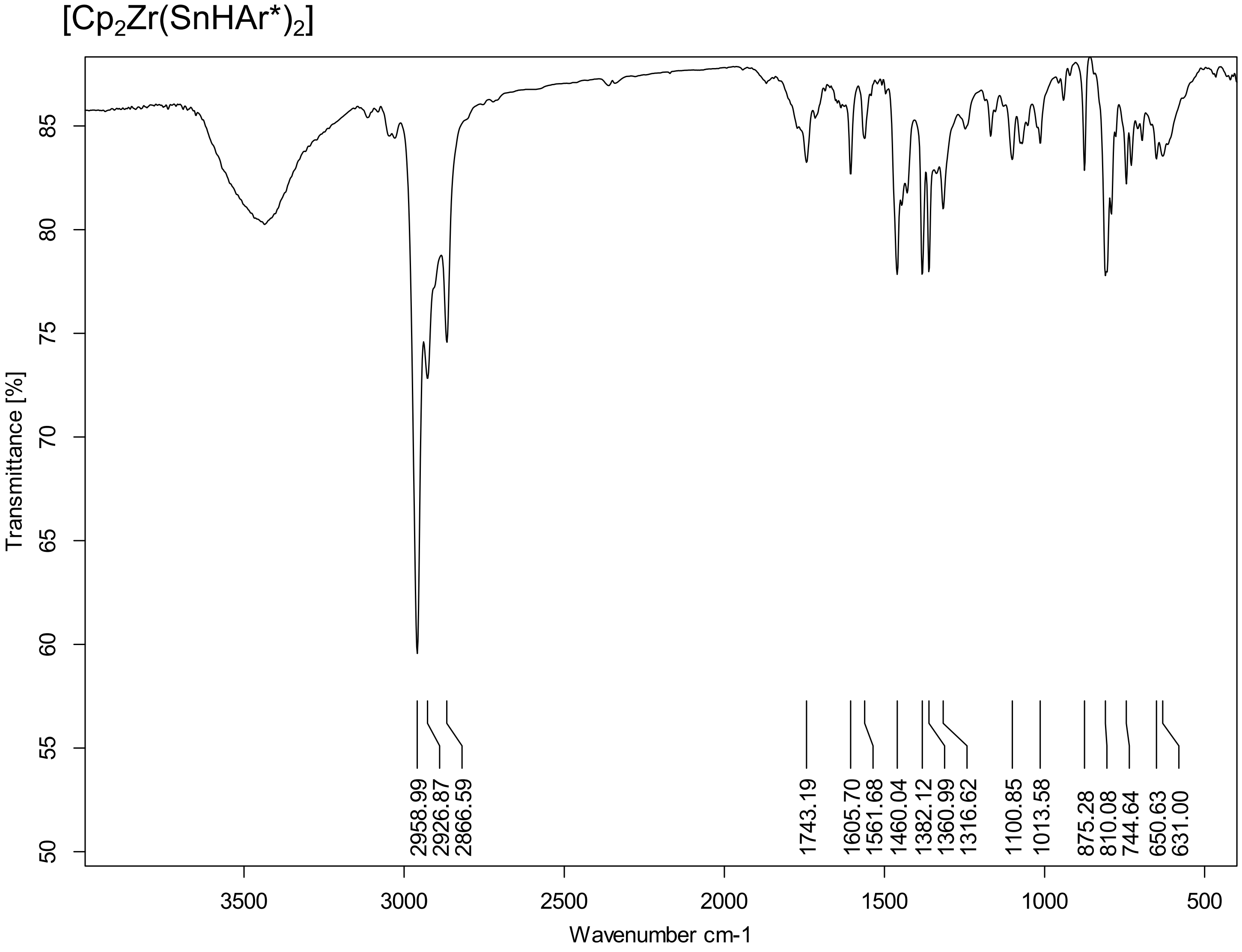

Figure S20. IR spectrum of **5**.

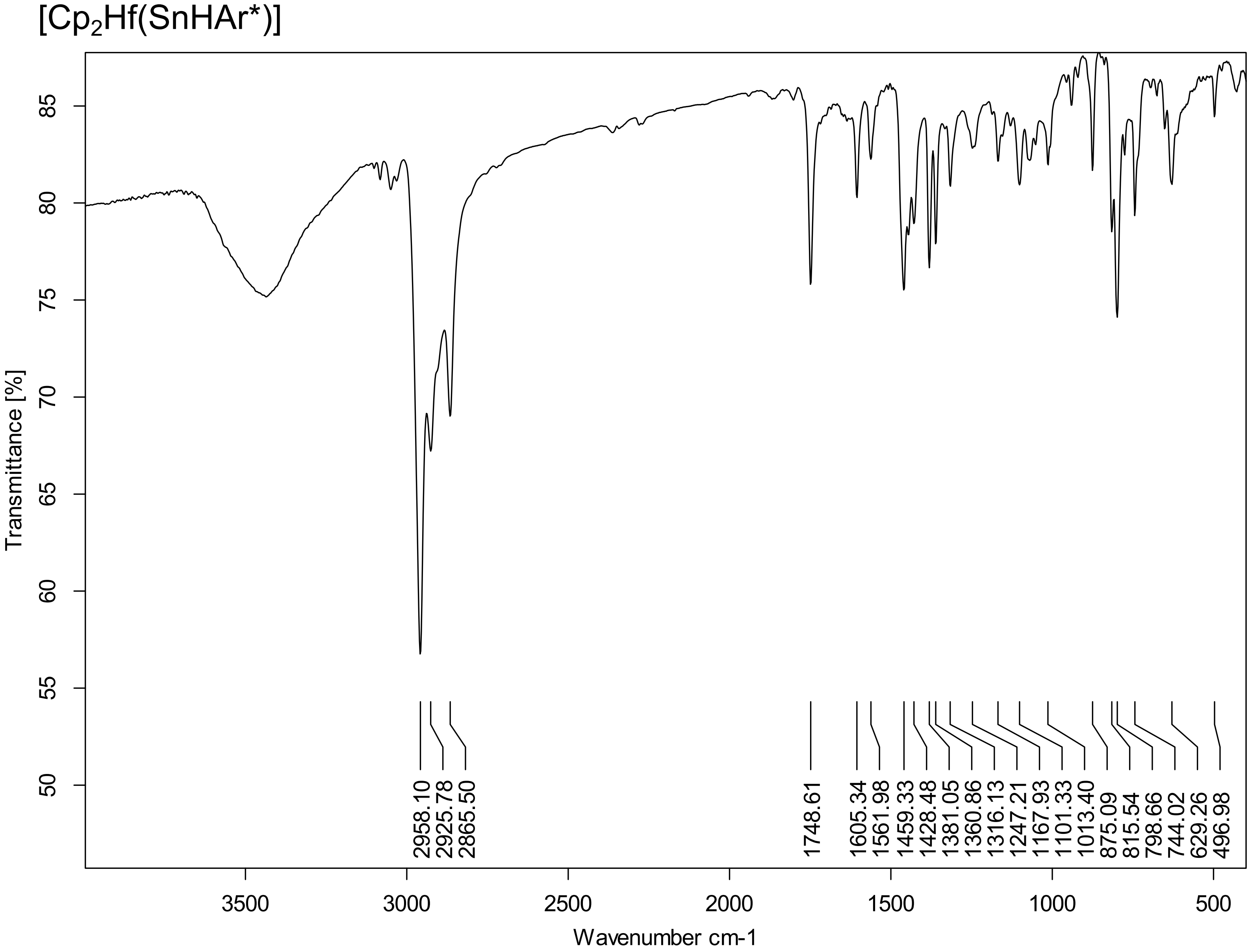

Figure S21. IR spectrum of **6**.

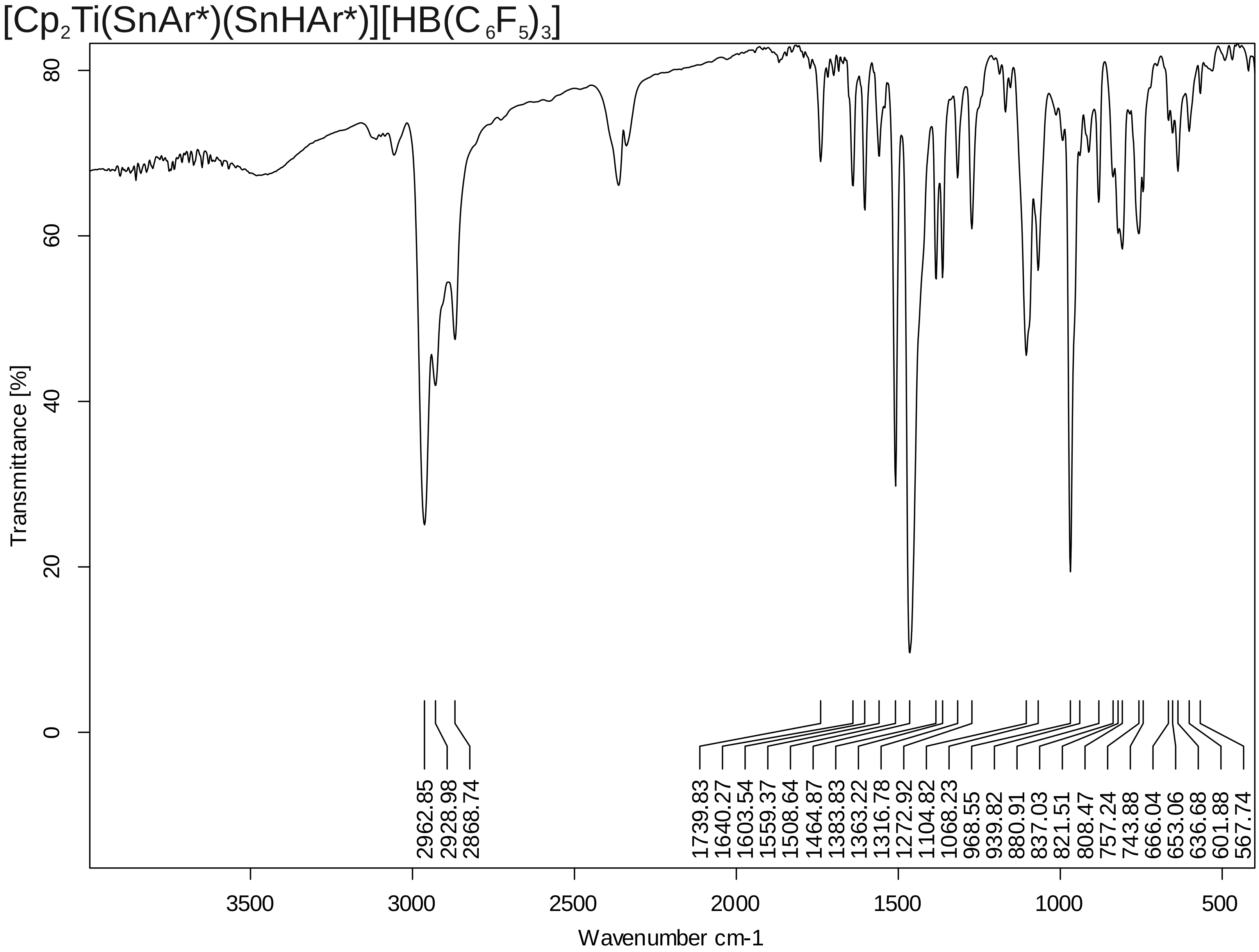

Figure S22. IR spectrum of **7**.

5. Quantum chemical calculations

DFT calculations were carried out with Gaussian09.[3] Natural bond orbitals were obtained using the NBO 6.0 software,[4] and the topology of the electron density was analysed employing the software package AIMALL.[5] Plots were generated with the software Chemcraft.[6] NMR calculations were carried out using ADF with the GGA revPBE-D3(BJ) functional and ZORA TZ2P basis set.[7] Relativistic effects were taken into account by the ZORA[8] method using the spin-orbit coupling option.

The geometry of $[Cp_2Ti(SnHAr^*)_2]$, **4** (Ar* = 2,6-$Trip_2C_6H_3$, Trip = 2,4,6-triisopropylphenyl) was optimized using the BP86 density functional along with the implemented def2TZVP basis set for all atoms, except Ti and Sn.[9] For these atoms, Stuttgart Dresden effective core potentials were employed, in combination with optimised valence basis sets as implemented in Gaussian 09.[10] Dispersion corrections were included by adding the D3 version of Grimme´s dispersion with Becke-Johnson damping.[11]

The geometry of the model systems $[Cp_2Zr(SnHPh)_2]$ and $[Ar^*SnH(^{Et}NHC)]$ were optimised using the B3LYP functional,[12] along with the TZVP[13] basis set for all atoms, except Sn and Zr. For these atoms, Stuttgart Dresden effective core potentials were employed, in combination with optimised valence basis sets.[9, 10b, 12a, 14] Dispersion corrections were included by adding the D3 version of Grimme´s dispersion with Becke-Johnson damping.[11, 15]

The geometry of the cationic complex $[Cp_2Ti(SnAr^*)(SnHAr^*)]^+$, **7** (Ar* = 2,6-$Trip_2C_6H_3$, Trip = 2,4,6-triisopropylphenyl) was optimised using the M062X functional,[16] [16] along with the implemented 6-311G(d,p)[17] basis set for atoms H, C and Ti. A Stuttgart Dresden effective core potential was employed for Sn,[10b] in combination with an optimised valence basis set as implemented in Gaussian 09.

All geometry optimisations were performed without imposing any symmetry constraints and the structures were confirmed as true minima by calculating analytical frequencies. Only in the case of **7**, one spurious frequency (-1.18 cm^{-1}) was observed.

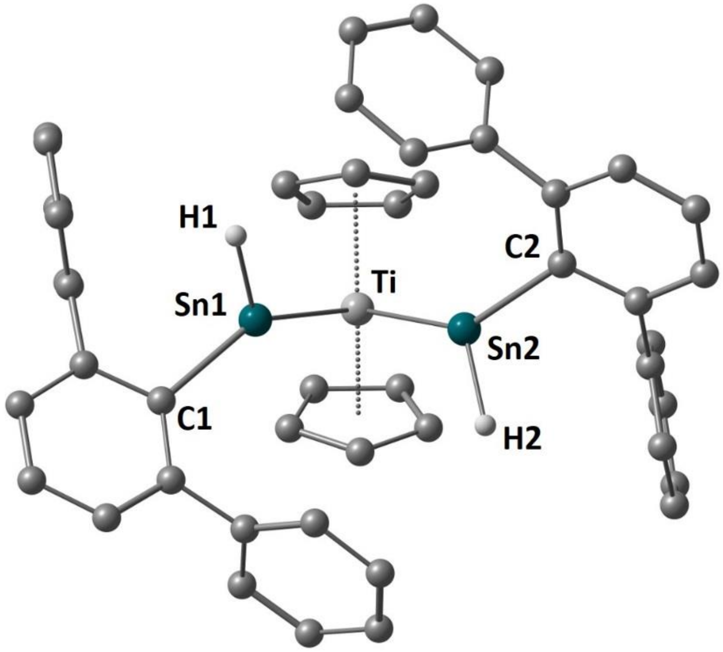

Figure S23. Optimized DFT geometry of $Cp_2Ti(SnHAr^*)_2$, **4** (Ar* = 2,6-$Trip_2C_6H_3$, Trip = 2,4,6-triisopropylphenyl). *i*Pr substituents and all hydrogen atoms attached to carbon atoms are omitted for clarity. Selected distances [Å] and angles [deg]: Ti–Sn1 2.671, Ti–Sn2 2.674, Sn1–Sn2 3.474, Sn1–H1 1.754, Sn2–H2 1.754, Sn1–C1 2.219, Sn2–C2 2.220, Sn1–Ti–Sn2 81.1, C1–Sn1–Ti 140.9, C2–Sn2–Ti 140.9.

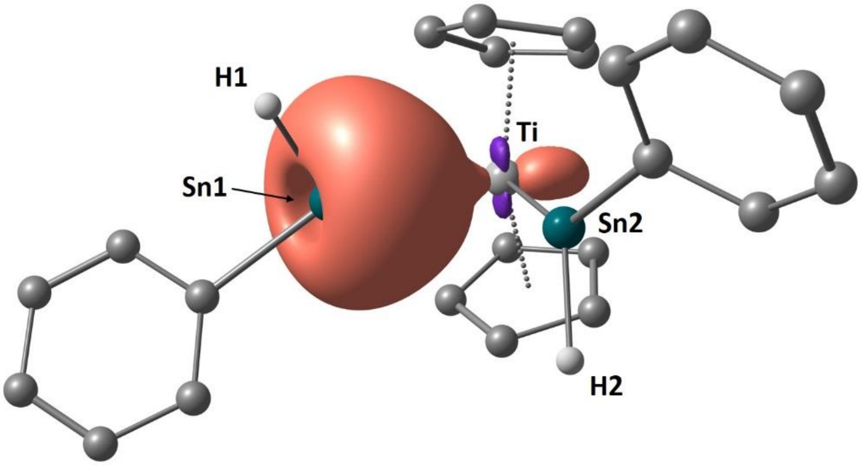

Figure S24. NBO 93 of $Cp_2Ti(SnHAr^*)_2$, **4**, representing the σ bond between Sn1 and Ti. Occupation: 1.85077; 42.1 % Ti (15.7 % s, 84.2 % d character) and 57.9 % Sn (53.6 % s, 46.4 % p character). All aromatic substituents and hydrogen atoms attached to carbon atoms are omitted for clarity. NBO 96 (not shown) represents the analogous σ bond between Sn2 and Ti [occupation: 1.85018; 41.9 % Ti (15.7 % s, 84.2 % d character) and 58.1 % Sn (53.7 s, 46.4 % p)].

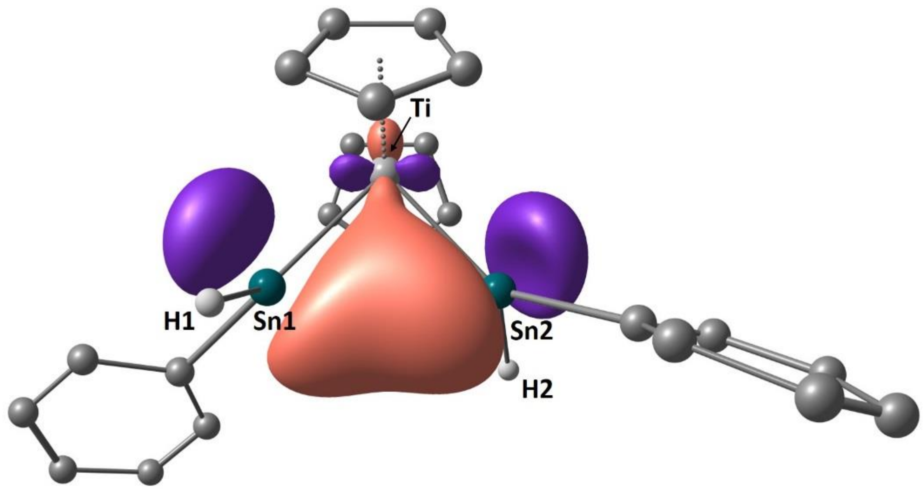

Figure S25. 3-centre NBO 311 of $Cp_2Ti(SnHAr^*)_2$, **4**, representing the π bond between Ti and both Sn1 and Sn2. Occupation: 1.72749; 47.7 % Ti (2.9 % s, 97.0 % d character), 26.2 % Sn1 (99.4 % p character) and 26.2 % Sn2 (99.4 % p character). All aromatic substituents and hydrogen atoms attached to carbon atoms are omitted for clarity.

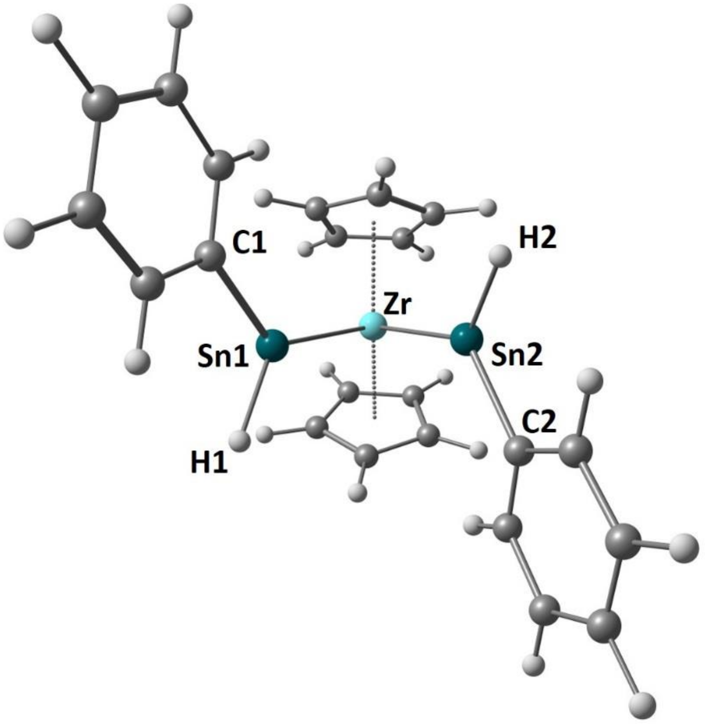

Figure S26. Optimized DFT geometry of the model system $Cp_2Zr(SnHPh)_2$. Selected distances [Å] and angles [deg]: Zr–Sn1 2.827, Zr–Sn2 2.827, Sn1–Sn2 3.273, Sn1–H1 1.740, Sn2–H2 1.740, Sn1–C1 2.168, Sn2–C2 2.168, Sn1–Zr–Sn2 70.7, C1–Sn1–Zr 132.6, C2–Sn2–Zr 132.6.

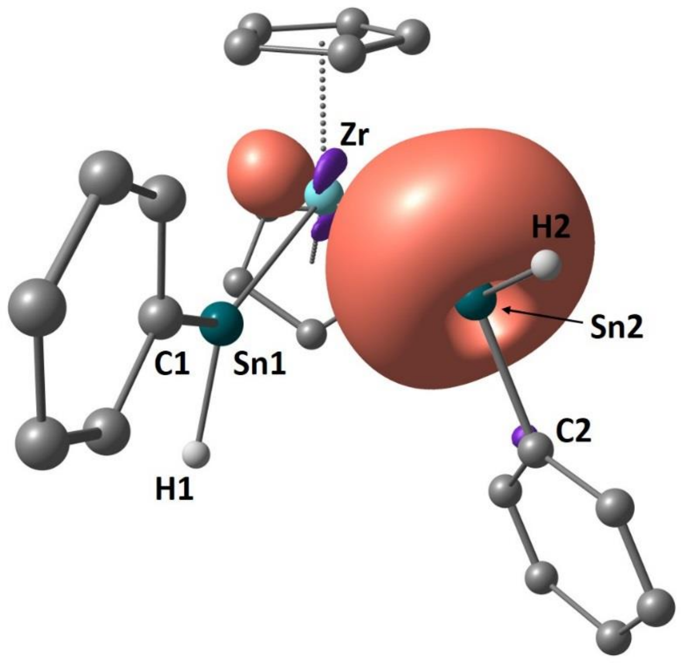

Figure S27. NBO 84 of the model system $Cp_2Zr(SnHPh)_2$, representing the σ bond between Sn2 and Zr. Occupation: 1.91575; 36.6 % Zr (16.6 % s, 83.1 % d character) and 63.4 % Sn (48.4 % s, 51.4 % p character). All hydrogen atoms attached to carbon atoms are omitted for clarity. NBO 37 (not shown) represents the analogous σ bond between Sn1 and Ti [occupation: 1.91575; 36.6 % Zr (16.6 % s, 83.1 % d character) and 63.4 % Sn (48.4 s, 51.4 % p)].

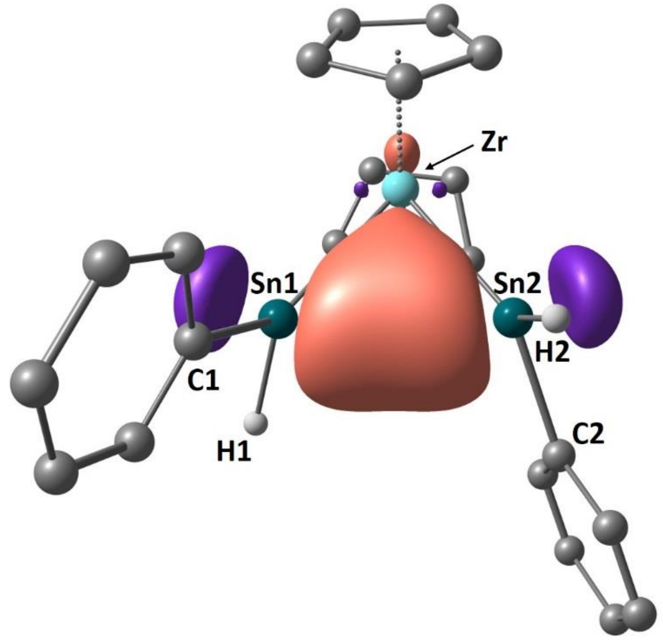

Figure S28. 3-centre NBO 87 of the model system $Cp_2Zr(SnHPh)_2$, representing the π bond between Zr and both Sn1 and Sn2. Occupation: 1.84963; 37.3 % Zr (2.8 % s, 97.0 % d character), 31.4 % Sn1 (2.1 % s, 97.6 % p character) and 31.4 % Sn2 (2.1 % s, 97.6 % p character). All hydrogen atoms attached to carbon atoms are omitted for clarity.

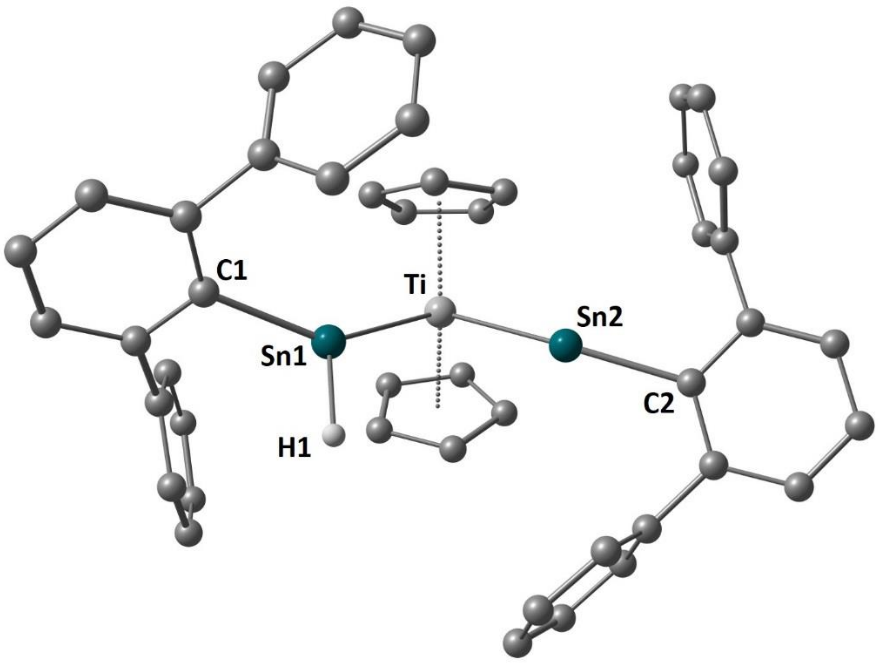

Figure S29. Optimized DFT geometry of $Cp_2Ti(SnAr^*)(SnHAr^*)$, **7** (Ar* = 2,6-$Trip_2C_6H_3$, Trip = 2,4,6-triisopropylphenyl). *i*Pr substituents and all hydrogen atoms attached to carbon atoms are omitted for clarity. Selected distances [Å] and angles [deg]; values from X-ray diffraction are denoted in square brackets: Ti–Sn1 2.762 [2.6886(7)], Ti–Sn2 2.578 [2.5644(7)], Sn1–H1 1.745 [1.887(17)], Sn1–C1 2.172 [2.176(4)], Sn2–C2 2.154 [2.142(3)], Sn1–Sn2 3.913 [3.925(1)], C1–Sn1–Ti 140.1 [145.0(1)], C2–Sn2–Ti 150.3 [167.4(1)], Sn1–Ti–Sn2 94.2 [96.7(1)].

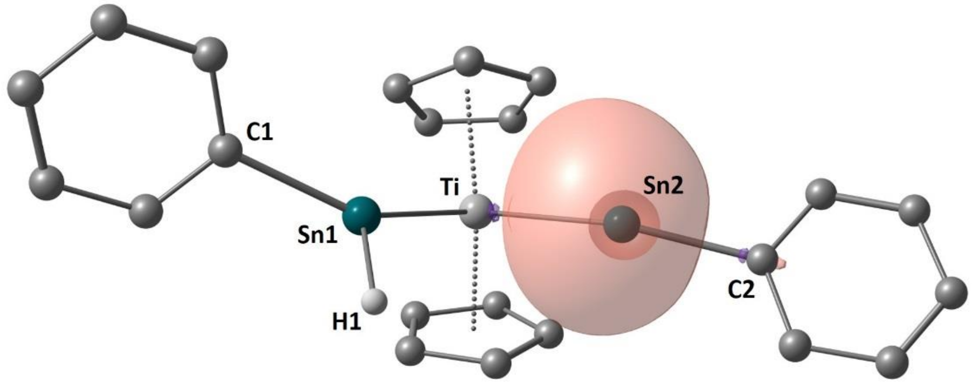

Figure S30. NBO 94 of $Cp_2Ti(SnAr^*)(SnHAr^*)$, **7**, representing the lone pair on Sn2, which is donated towards Ti to form a σ-bond. Occupation: 1.65247. All aromatic substituents and hydrogen atoms attached to carbon atoms are omitted for clarity. Composition of the corresponding NLMO 94 (not shown), which includes the delocalization towards the Ti centre: Occupation: 2.00000; 81.4 % Sn2 (88.4 % s, 11.6 % p character) and 11.8 % Ti (32.7 % s, 67.1 % d character).

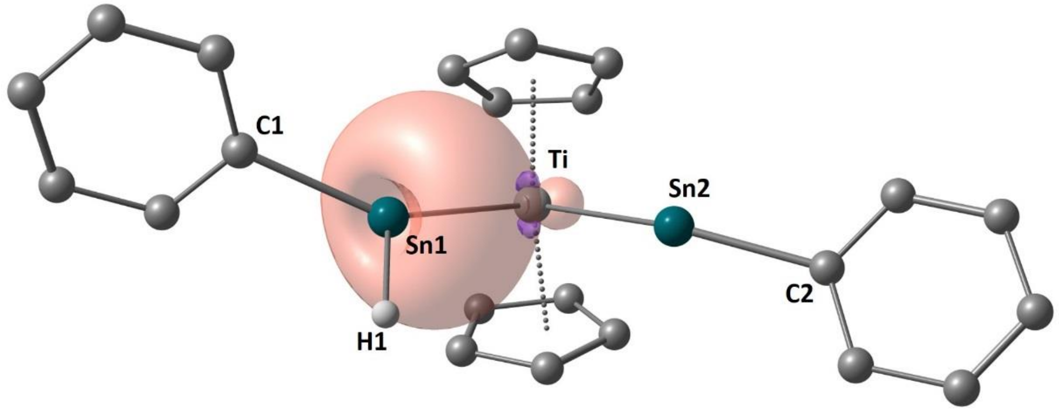

Figure S31. NBO 313 of $Cp_2Ti(SnAr^*)(SnHAr^*)$, **7**, representing the σ bond between Sn1 and Ti. Occupation: 1.82661; 26.4 % Ti (25.1 % s, 74.7 % d character) and 73.6 % Sn (48.6 % s, 51.4 % p character). All aromatic substituents and hydrogen atoms attached to carbon atoms are omitted for clarity.

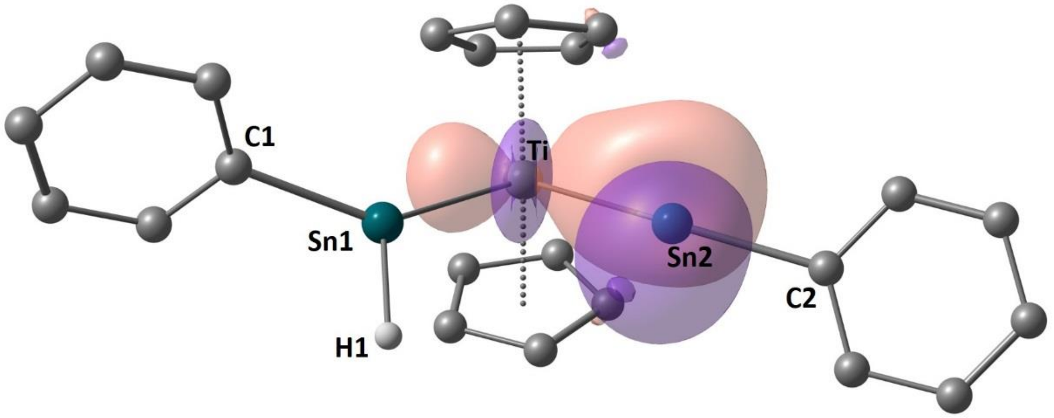

Figure S32. NBO 315 of $Cp_2Ti(SnAr^*)(SnHAr^*)$, **7**, representing the π bonding between Ti and Sn2. Occupation: 1.6593; 68.8 % Ti (2.1 % s, 97.8 % d character) and 31.2 % Sn2 (2.4 % s, 97.6 % p character). All aromatic substituents and hydrogen atoms attached to carbon atoms are omitted for clarity.

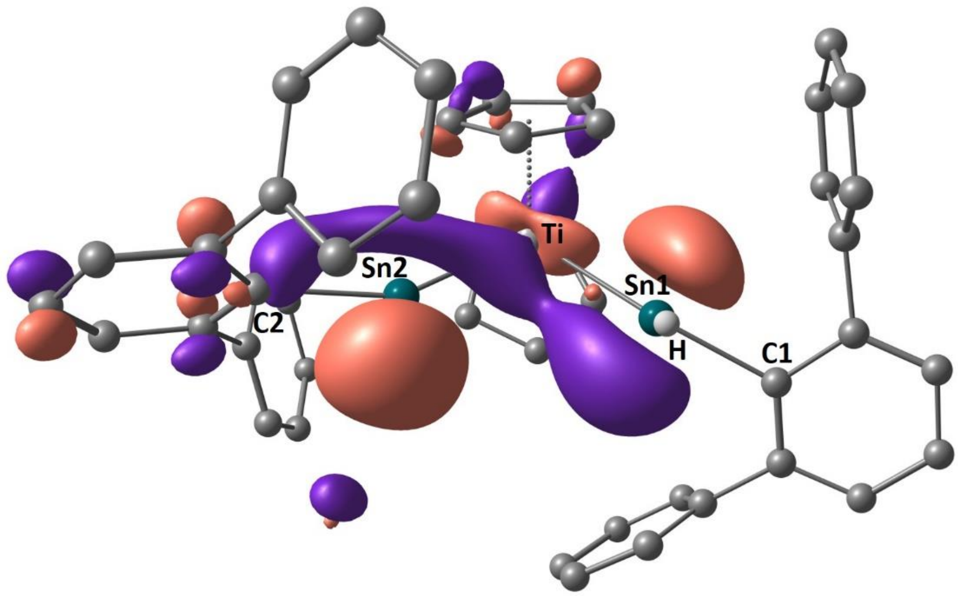

Figure S33. LUMO of $Cp_2Ti(SnAr^*)(SnHAr^*)$, **7**.

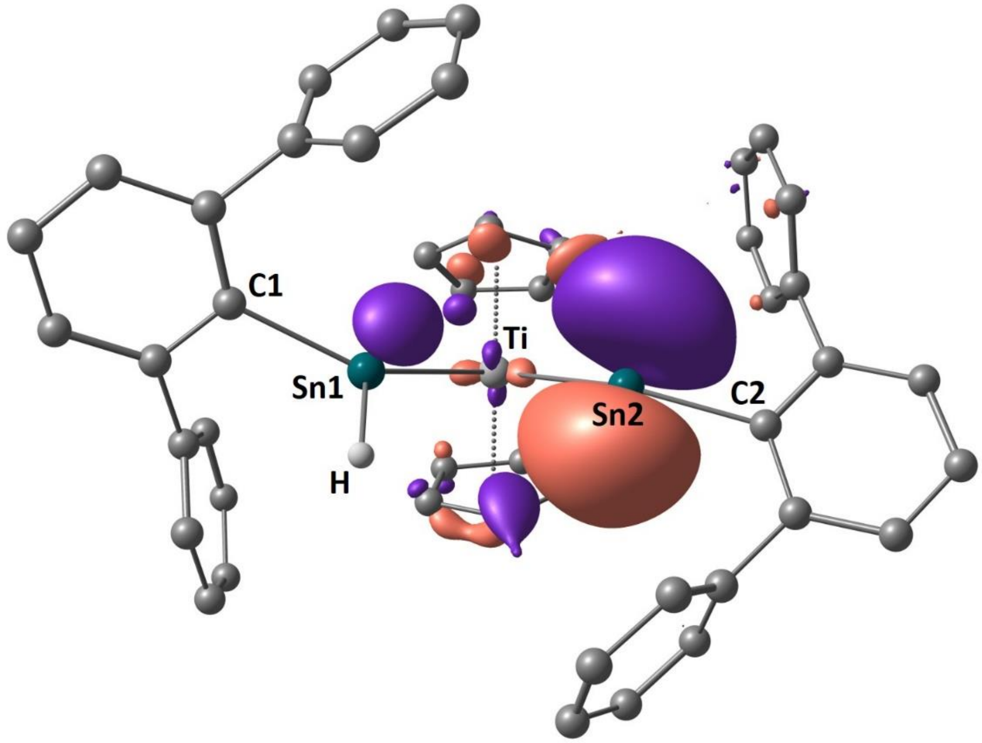

Figure S34. LUMO+1 of $Cp_2Ti(SnAr^*)(SnHAr^*)$, **7**.

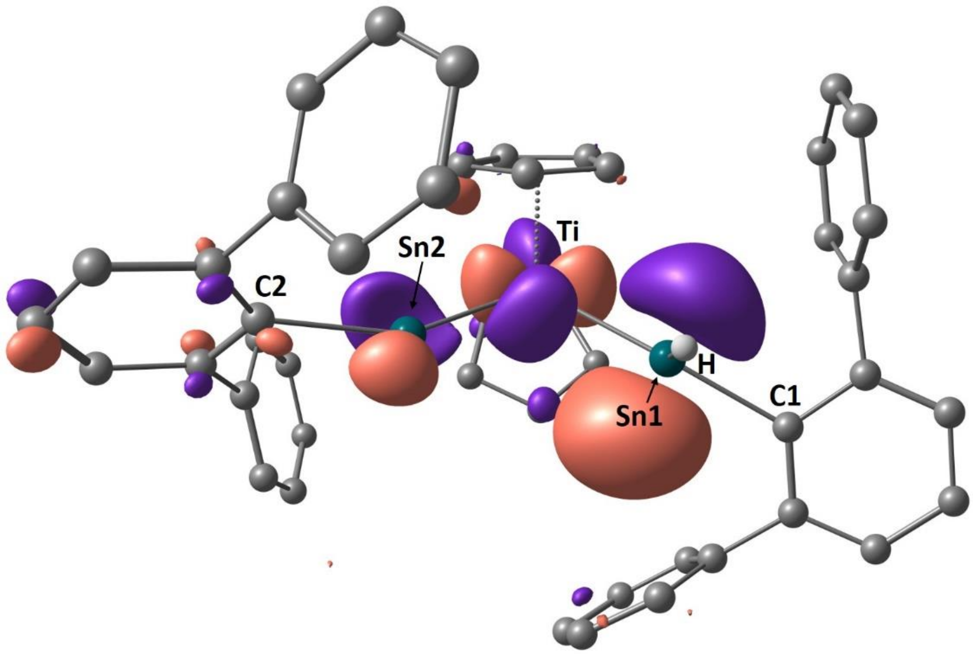

Figure S35. LUMO+2 of $Cp_2Ti(SnAr^*)(SnHAr^*)$, **7**.

6. References

[1] J.-J. Maudrich, F. Diab, S. Weiß, M. Widemann, T. Dema, H. Schubert, K. M. Krebs, K. Eichele, L. Wesemann, *Inorg. Chem.*, doi.org/10.1021/acs.inorgchem.1029b01822.

[2] a) G. M. Sheldrick, **SADABS, 2008 University of Göttingen, Göttingen, Germany**; b) L. Farrugia, *J. Appl. Crystallogr.* **2012**, *45*, 849-854; c) C. B. Hübschle, G. M. Sheldrick, B. Dittrich, *J. Appl. Crystallogr.* **2011**, *44*, 1281-1284; d) Bruker AXS Inc., SAINT, APEX2, Madison, Wisconsin, USA, **2012**.

[3] M. J. Frisch, G. W. Trucks, H. B. Schlegel, G. E. Scuseria, M. A. Robb, J. R. Cheeseman, G. Scalmani, V. Barone, B. Mennucci, G. A. Petersson, H. Nakatsuji, M. Caricato, X. Li, H. P. Hratchian, A. F. Izmaylov, J. Bloino, G. Zheng, J. L. Sonnenberg, M. Hada, M. Ehara, K. Toyota, R. Fukuda, J. Hasegawa, M. Ishida, T. Nakajima, Y. Honda, O. Kitao, H. Nakai, T. Vreven, J. J. A. Montgomery, J. E. Peralta, F. Ogliaro, M. Bearpark, J. J. Heyd, E. Brothers, K. N. Kudin, V. N. Staroverov, R. Kobayashi, J. Normand, K. Raghavachari, A. Rendell, J. C. Burant, S. S. Iyengar, J. Tomasi, M. Cossi, N. Rega, J. M. Millam, M. Klene, J. E. Knox, J. B. Cross, V. Bakken, C. Adamo, J. Jaramillo, R. Gomperts, R. E. Stratmann, O. Yazyev, A. J. Austin, R. Cammi, C. Pomelli, J. W. Ochterski, R. L. Martin, K. Morokuma, V. G. Zakrzewski, G. A. Voth, P. Salvador, J. J. D. S. Dapprich, A. D. Daniels, Ö. Farkas, J. B. Foresman, J. V. Ortiz, J. Cioslowski, D. J. Fox, *Gaussian 09, Revision D.01, Gaussian, Inc., Wallingford CT* **2009**.

[4] a) E. D. Glendening, C. R. Landis, F. Weinhold, *J. Comput. Chem.* **2013**, *34*, 1429-1437; b) E. D. Glendening, J. K. Badenhoop, A. E. Reed, J. E. Carpenter, J. A. Bohmann, C. M. Morales, C. R.

Landis, F. Weinhold, *Theoretical Chemistry Institute, University of Wisconsin, Madison* **2013**; c) A. E. Reed, R. B. Weinstock, F. Weinhold, *J. Chem. Phys.* **1985**, *83*, 735-746.

[5] T. A. Keith, TK Gristmill Software, Overland Park KS, USA, **2017**.

[6] G. A. Zhurko, CHEMCRAFT (http://www.chemcraftprog.com).

[7] Vrije Universiteit, Amsterdam, The Netherlands, htpp://www.scm.com.

[8] a) S. K. Wolff, T. Ziegler, *J. Chem. Phys.* **1998**, *109*, 895-905; b) S. K. Wolff, T. Ziegler, E. van Lenthe, E. J. Baerends, *J. Chem. Phys.* **1999**, *110*, 7689-7698.

[9] a) J. P. Perdew, *Phys. Rev. B* **1986**, *33*, 8822-8824; b) F. Weigend, R. Ahlrichs, *Phys. Chem. Chem. Phys.* **2005**, *7*, 3297-3305; c) F. Weigend, *Phys. Chem. Chem. Phys.* **2006**, *8*, 1057-1065.

[10] a) M. Dolg, U. Wedig, H. Stoll, H. Preuss, **1987**, *86*, 866-872; b) A. Bergner, M. Dolg, W. Küchle, H. Stoll, H. Preuß, *Mol. Phys.* **1993**, *80*, 1431-1441.

[11] a) S. Grimme, J. Antony, S. Ehrlich, H. Krieg, *J. Chem. Phys.* **2010**, *132*, 154104; b) S. Grimme, S. Ehrlich, L. Goerigk, *J. Comput. Chem.* **2011**, *32*, 1456-1465.

[12] a) A. D. Becke, *Phys. Rev. A* **1988**, *38*, 3098-3100; b) C. Lee, W. Yang, R. G. Parr, *Phys. Rev. B* **1988**, *37*, 785-789; c) S. H. Vosko, L. Wilk, M. Nusair, *Can. J. Phys.* **1980**, *58*, 1200-1211; d) P. J. Stephens, F. J. Devlin, C. F. Chabalowski, M. J. Frisch, *J. Phys. Chem.* **1994**, *98*, 11623-11627; e) A. D. Becke, *J. Chem. Phys.* **1993**, *98*, 5648-5652.

[13] A. Schäfer, C. Huber, R. Ahlrichs, *J. Chem. Phys.* **1994**, *100*, 5829-5835.

[14] a) D. Andrae, U. Häussermann, M. Dolg, H. Stoll, H. Preuß, *Theoret. Chim. Acta* **1990**, *77*, 123-141; b) J. M. L. Martin, A. Sundermann, *J. Chem. Phys.* **2001**, *114*, 3408-3420.

[15] S. Grimme, J. Antony, S. Ehrlich, H. Krieg, *J. Chem. Phys.* **2010**, *132*, 154104-154119.

[16] Y. Zhao, D. G. Truhlar, *Theor Chem Acta* **2008**, *120*, 215-241.

[17] a) M. J. Frisch, J. A. Pople, J. S. Binkley, *J. Chem. Phys.* **1984**, *80*, 3265-3269; b) R. Krishnan, J. S. Binkley, R. Seeger, J. A. Pople, *J. Chem. Phys.* **1980**, *72*, 650-654; c) A. J. H. Wachters, *J. Chem. Phys.* **1970**, *52*, 1033-1036.
